# Two new mite species of the genus *Zygoseius* Berlese from Mexico (Acari, Mesostigmata)

**DOI:** 10.3897/zookeys.629.10121

**Published:** 2016-11-07

**Authors:** Ali Ahadiyat, Frédéric Beaulieu

**Affiliations:** 1 Department of Entomology, Science and Research Branch, Islamic Azad University, Tehran, Iran; 2 Canadian National Collection of Insects, Arachnids and Nematodes, Agriculture and Agri-Food Canada, 960 Carling avenue, Ottawa, ON K1A 0C6, Canada

**Keywords:** Gamasina, Pachylaelapidae, taxonomy, Chiapas, North America

## Abstract

Two new species of mites of the genus *Zygoseius* Berlese, *Zygoseius papaver* **sp. n.** and *Zygoseius lindquisti* **sp. n.**, collected from moss and flood debris, respectively, in a creek in Chiapas State, Mexico, are described herein.

## Introduction

The genus *Zygoseius* Berlese, 1916 is a moderately small genus of mesostigmatic mites, with 13 described species currently. It was first defined by Berlese (1916) as a subgenus of *Lasioseius* Berlese, 1916, with description of the species *Zygoseius furciger*, collected from ants’ nests in Argentina. The genus was variously reviewed by Halliday (1997), Karg (1998) and Karg and Schorlemmer (2009). *Zygoseius* species are found in soil, leaf litter, moss, compost, cow and chicken dung, and ants’ nests (Halliday 1997, Karg 1998, Karg and Schorlemmer 2009). Some species were found in association with insects, namely dung beetles (e.g. *Zygoseius furciger* (Costa 1963) and *Zygoseius sarcinulus* Halliday, 1997 (Halliday 1997)). Feeding behavior has been observed for one species, *Zygoseius furciger*, which fed readily on nematodes (Walter and Ikonen 1989).

The taxonomic placement of *Zygoseius* is still problematic and authors placed it in various families: Ascidae *sensu lato* or Blattisociidae (Evans 1958, Sheals 1962, Costa 1963, Hyatt 1964), Halolaelapidae (Karg 1998, Christian and Karg 2006, Karg and Schorlemmer 2009), Laelapidae (Vitzthum 1943) and Pachylaelapidae (Lindquist and Evans 1965, Hafez and Nasr 1982, Krantz and Ainscough 1990, Halliday 1997, Moraza and Peña 2005, Lindquist et al. 2009, Childers and Ueckermann 2015). Mašán and Halliday (2014) excluded the genus from Pachylaelapidae based on its leg chaetotaxy and the two dorsal shields of the deutonymphs. Recently, the molecular analyses of Sourassou et al. (2015) suggest that *Zygoseius* is related to members of the superfamily Rhodacaroidea.

## Materials and methods

Mite specimens were collected from moss and debris in Chiapas State (officially the Free and Sovereign State of Chiapas), Mexico, in May 1969. All specimens had been extracted from samples using Berlese-Tullgren funnels, then cleared in lactophenol and mounted in Hoyer’s medium on microscope slides. Specimens were examined using a Zeiss Axio Imager M2 and a Leica DM 2500 compound scopes, attached to cameras AxioCam ICc 5 and ICC50 HD, respectively. Images and morphological measurements were taken via ZEN 2012 software (version 8.0) and Leica Application Suite (LAS) software (version 4.2, Live and Interactive Measurements modules). More than 120 morphological characters were examined and measured for each species. All the measurements were given as ranges of minimum–maximum, in micrometers (µm). Lengths of shields were taken along their midlines from the anterior to posterior margins; widths were measured approximately at mid-level (at the widest point) for the dorsal shield, between mid-level of coxae II (at the narrowest point) for the female sternal shield, and from the posterior part of coxae IV (at the widest point) for the male holoventral shield. Epigynal shield lengths were measured along their midlines from anterior margin of hyaline extension to posterior shield margin and also from the level of setae *st5* to the posterior shield margin. Epigynal and ventrianal shield widths were measured at the widest point, past *st5* level, and near *ZV2* level, respectively. Leg lengths were measured ventromedially from the base of coxa to the apex of tarsus, excluding the ambulacrum (ambulacral stalk, claws and pulvillus); lengths of leg segments were taken dorsomedially. Ambulacra were measured ventromedially including pulvilli and claws. Setae lengths were measured from the bases of their insertions to their tips. Distances between setae were measured from the center of the setal alveolae. Corniculi were measured from the apex to the median section of posterior margins. Chelicera lengths were measured for: the first or basal segment, second segment (from base to apex of the fixed digit; width measured at the widest point), fixed digit (from dorsal poroid to apex) and movable digit (from base to apex). Length of peritreme was measured from the anterior margin of stigmata to the anterior end of peritreme. Length and width of anal opening were measured excluding the raised band of cuticle surrounding the anus. Idiosomal notation for setae used in this paper follows that of Lindquist and Evans (1965). The notations for leg and palp setae follow those of Evans (1963a, 1963b). Idiosomal and peritrematal shield notations for pore-like structures (gland pores and poroids/lyrifissures) follow the systems of Athias-Henriot (1971) for ventral idiosoma and Athias-Henriot (1975) for dorsal idiosoma. The notations of spermathecal structures are based on Athias-Henriot (1968) and Evans and Purvis (1987).

## Results

### Zygoseius papaver sp. n.

Taxon classificationAnimaliaMesostigmataPachylaelapidae

http://zoobank.org/0DFF7672-E02A-48B3-90D8-1CC9D5601100

Figures 1 , 2 , 3 , 4 , 5 , 6 , 7 , 8 , 9 , , 10–13 , 14 , 27 , 28 , 29 , 30 , 31 , Plate 1

#### Diagnosis

**(female).** Dorsal shield oval, well-reticulated throughout, except nearly smooth medially between setae *j6*–*J4*; shield with serrated lateral margins. Dorsal setae smooth, relatively short, all <35 long, some podonotal (*s3*–*5*, *z6*) and opisthonotal (*J1*, *J2*, *J4*, *Z1*–*4*) setae longer than other setae; setae *J5* strongly mesad, and slightly anterad *Z5*. Sternal shield irregularly and sparsely micropunctate, with a transverse, recurved linea posterad level of setae *st1*. Epigynal shield punctate, mostly anteriorly and laterally. Ventrianal shield wider than long, lineate except anterad anus, and punctate except in anterior fourth; setae *JV1*–*2* 1.5–2× as long as other setae on shield. Peritrematal shield micropunctate; punctae larger in poststigmatic region. Soft lateral and opisthogastric integument bearing nine pairs of short setae. Epistome bifurcate, distal haves of projections bipectinate. Hypostomal setae *h1* twice as long as *h2* and 1.5× as long as *h3*. Cheliceral movable digit with two subapical, unconspicuous teeth. Cheliceral fixed digit with two subapical teeth. Genua II–III with 10 and 8 setae, lacking setae *av* and *pv*, respectively. Spermathecal apparatus with globular spermatheca separated from small, ring-like sperm reservoir by a thick-walled, short duct; spermatic canal long, narrow.

#### Description.

**Figure 1.**
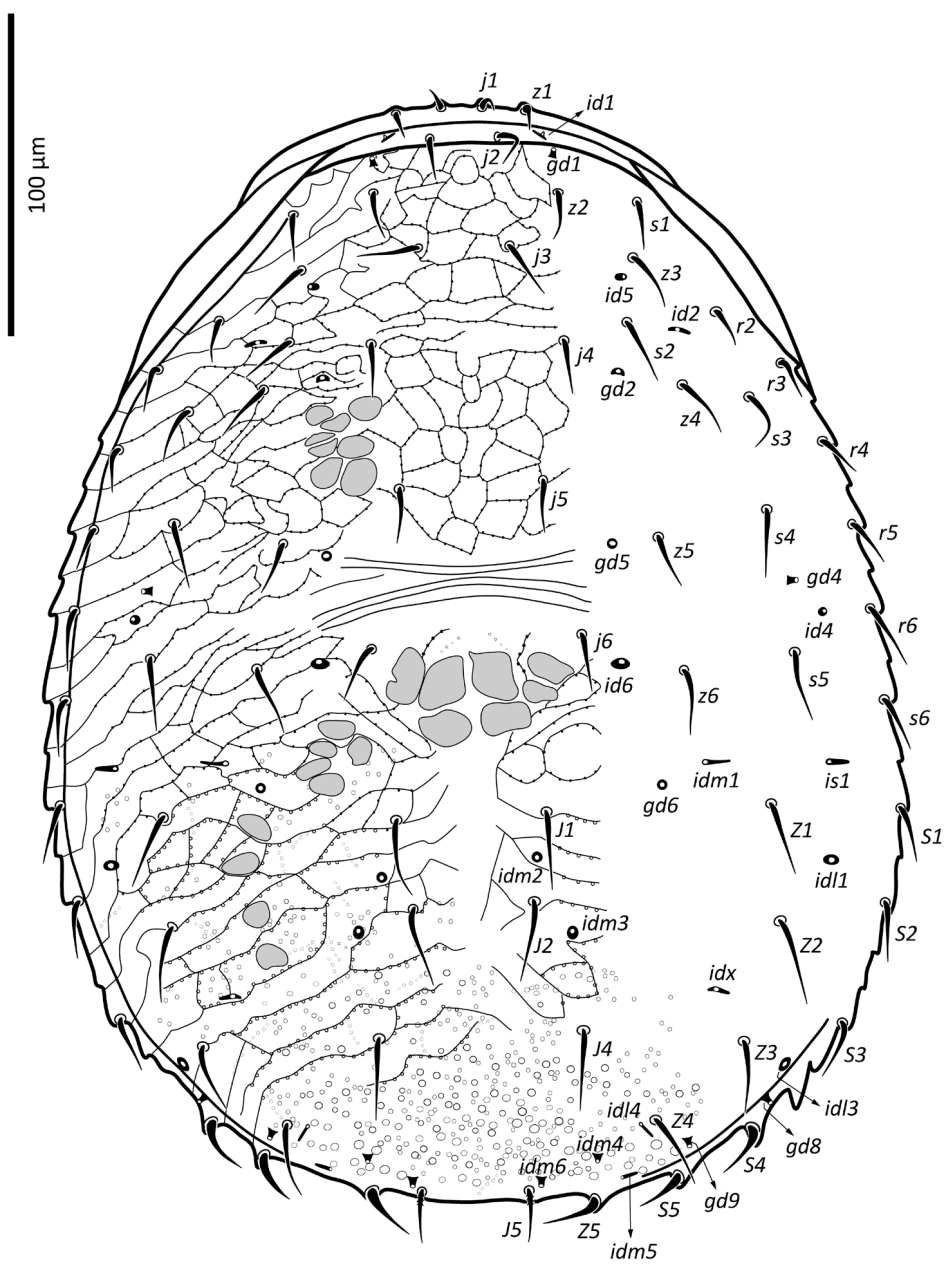
*Zygoseius papaver* sp. n., female, dorsal idiosoma.

*Female* (n = 11). *Dorsal idiosoma* (Figs 1, 28). Dorsal shield ovoid, 340–374 long, 252–275 wide (length/width ratio: 1.26–1.44), completely covering idiosoma, slightly widened posteriorly. Shield margins serrated posterolaterally from level of setae *r3*. Shield well-reticulated throughout, except more or less smooth medially in *j5–6* region and in median narrow band between setae *j6*–*J4*. Reticulations in opisthonotal region densely covered with small punctae. Posterior region between pairs of setae *J4*, *Z4*, *J5* with large punctae, not reticulate. Dorsal shield bearing 37 pairs of setae, 23 and 14 pairs on podonotal and opisthonotal regions, respectively; setae *J3* missing. Dorsal setae less than 35 long (Table 1), all smooth, acuminate, slightly widened in basal halves, except *J5* pilose in basal half (Fig. 3A); setae *J4* slightly pilose basally in some specimens (Fig. 3B). Dorsal idiosoma with 23 pairs of pore-like structures, including seven gland openings and 16 poroids.

**Table 1.** Lengths of most idiosomal setae of *Zygoseius papaver* sp. n. and *Zygoseius lindquisti* sp. n.

Setae	*Zygoseius papaver*		*Zygoseius lindquisti*
	Female (n = 11)	Male (n = 1)	Female (n = 2)
***j1***	10–15	?	~ 5–7
***j2***	17–25	?	14–17
***j3***	19–27	24	15–17
***j4***	18–25	21	16–19
***j5***	16–20	~ 15	14–17
***j6***	16–20	17	16–20
***J1***	26–30	24	27–32
***J2***	24–32	26	28–34
***J4***	24–30	22–24	30–31
***J5***	16–22	17–19	19–21
***z1***	9–12	?	~ 5–7
***z2***	17–21	~ 12	13–18
***z3***	17–25	~ 20	17–19
***z4***	19–31	22	15–19
***z5***	16–23	16	15–19
***z6***	24–32	30	26–34
***Z1***	22–29	27	30–31
***Z2***	25–30	26	33–34
***Z3***	23–28	22	31–33
***Z4***	22–28	22	30–31
***Z5***	15–22	16	20–26
***s1***	12–17	~ 11	14–19
***s2***	19–26	?	17–22
***s3***	21–28	22	19–21
***s4***	22–27	25	18–21
***s5***	23–30	25	22–24
***s6***	19–22	18	28–29
***S1***	18–24	?	27–31
***S2***	17–23	18	29–32
***S3***	16–21	~ 16	26–30
***S4***	16–22	19	27–31
***S5***	16–21	17	28–31
***r2***	12–20	~ 16	19–20
***r3***	14–17	15	19–21
***r4***	18–20	21	20–21
***r5***	17–20	?	22–25
***r6***	19–20	~ 20	24–29
***st1***	16–21	18	16–20
***st2***	17–23	16	20–23
***st3***	17–22	18	18–21
***st4***	15–20	13	16–19
***st5***	18–24	14	18–19
***JV1***	25–32	25–27	19–23
***JV2***	26–34	28–30	22–25
***JV3***	16–22	17–18	16–19
***JV4***	13–17	13–14	20–21
***JV5***	14–18	14–15	18–19
***ZV1***	12–18	10–14	15–16
***ZV2***	11–17	12	18–21
***ZV3***	14–17	13–15	18–21
**Para-anal setae (*pa*)**	18–22	18	21–24
**Post-anal seta (*po*)**	17–23	16	20–22

? the seta was insufficiently clear to be measured.

**Table 2.** Distances between pairs of some dorsal and ventral idiosomal setae of *Zygoseius papaver* sp. n. and *Zygoseius lindquisti* sp. n.

Characters	*Zygoseius papaver*		*Zygoseius lindquisti*
	Female	Male	Female
***st1*–*st1***	31–41	37	41–48
***st2*–*st2***	43–47	41	50–53
***st3*–*st3***	39–45	45	50–54
***st4*–*st4***	51–57	37	61–63
***st5*–*st5***	55–62	39	62–65
***J1*–*J1***	37–49	31	52–58
***J4*–*J4***	63–80	61	81–83
***J4*–*J4*/*J1*–*J1***	1.38–1.72	1.96	1.42–1.57
***J2*–*J2***	34–47	38	45–47
***J1*–*J2***	26–35	31	36–41

**Figure 2.**
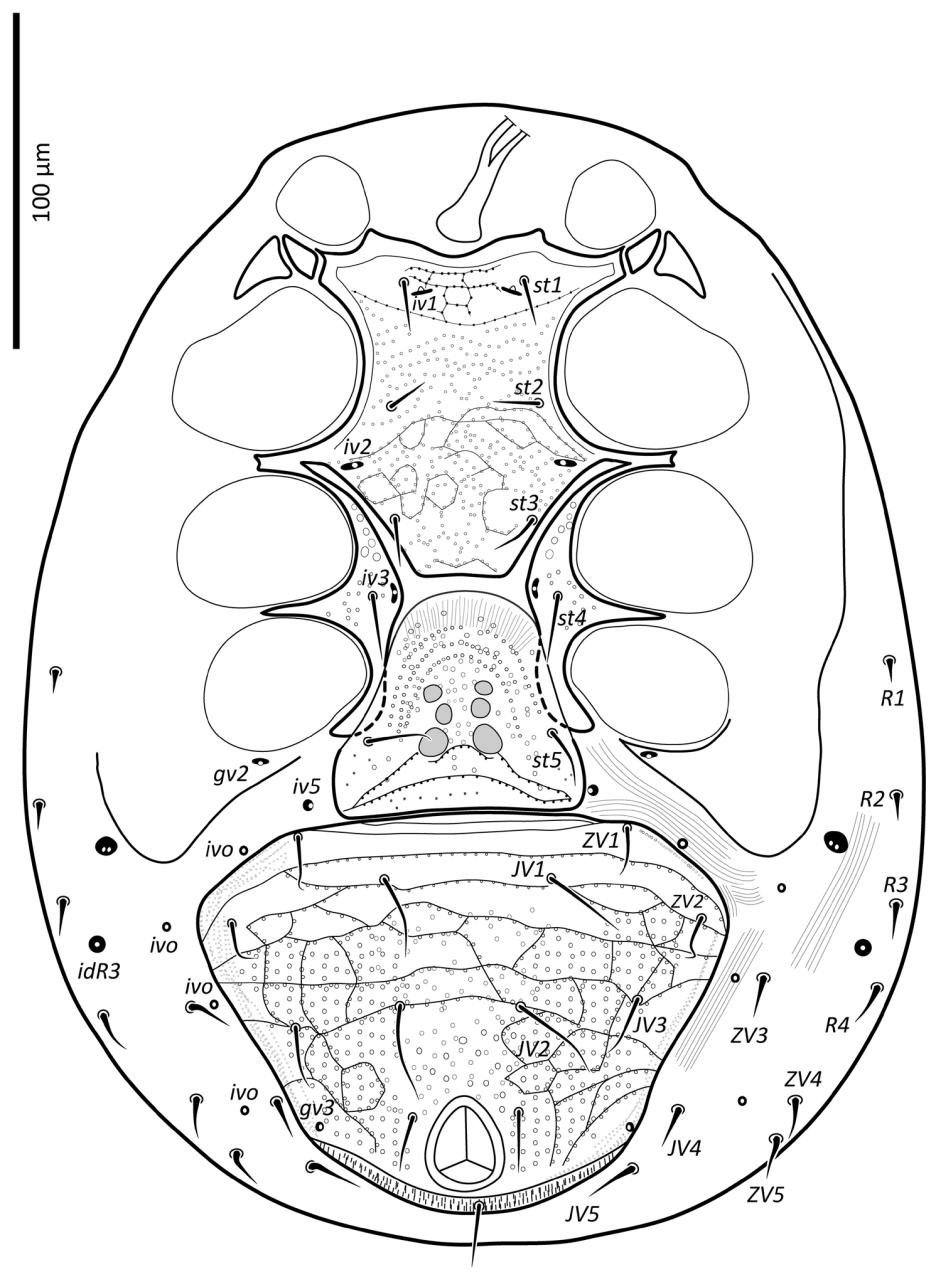
*Zygoseius papaver* sp. n., female, ventral idiosoma.

*Ventral idiosoma* (Figs 2, 29). Tritosternum with a trapezoidal base 22–27 long, 11–13 wide proximally, 4–6 wide apically, and a pair of laciniae, 76–83 long; laciniae with barbs relatively short and blunt (Fig. 4). Sternal shield 93–105 long, 55–65 wide (length/width ratio: 1.50–1.78), bearing two pairs of poroids (*iv1*–*2*), and three pairs of smooth, subequal setae *st1–3* (Table 1); anterolateral arms of shield each insensibly fragmented apically into a platelet, itself abutting subtriangular exopodal plate between coxae I and II; shield anterior margin with a weak, wide median depression and two subtriangular projections; posterior margin narrow, truncate. Shield irregularly and sparsely micropuntate. A transverse, recurved linea posterad level of setae *st1*. Metasternal platelets fused to endopodal elements, arc-like in shape, punctate, bearing simple setae *st4* and poroids *iv3*. Epigynal shield trapezoidal, 72–79 long, 22–27 long from *st5* to posterior margin, 68–81 wide (length/width ratio: 0.91–1.03), with punctae most conspicuous in anterior and lateral portions; lineate posteriorly, three pairs of large subcircular sigillae centrally; anterior hyaline portion rounded, poorly sclerotized, indistinct; shield widest past level of *st5*, with posterior margin truncate; closely abutting ventrianal shield. Setae *st5* smooth, inserted near shield lateral margins; poroids *iv5* near posterolateral margins of shield. Ventrianal shield subpentagonal, expanded, wider than long, 113–121 long, 147–180 wide (length/width ratio: 0.70–0.80), straight anteriorly between setae *ZV1*. Shield distinctly lineate anteriorly, distinctly punctate posteriorly and medially, weakly lineate posterad *JV2* level, with small punctae in lateral margins; shield with five pairs of pre-anal and three circum-anal setae, all smooth. Setae *JV1*–*2* subequal, 1.5–2× as long as other setae (Table 1); para-anal setae inserted near level of anterior margin of anal opening; gland openings *gv3* on posterolateral margins of shield near mid-level of anus; cribrum well-developed, with a few narrow transversal strips of spicules; anal opening 20–25 long, 18–22 wide, subtriangular to ovoid, located in posterior fourth or third of shield. Peritreme 175–198 long, densely covered by aciculae, extending anteriorly almost to level of seta *z1*, with one gland pore (*gp*) located at mid-level of coxa II. Peritrematal shield wide, essentially in ventral position; completely fused to exopodal, parapodal and metapodal elements, extending well behind posterior level of coxae IV. Shield essentially micropunctate throughout, with larger punctae in poststigmatic region, bearing four pore-like structures (*id3*, *gd3*, *id7*), including *gv2*. Exopodal element between coxae II–III insensibly separated from posterior portion of more posterior exopodal-peritrematal elements (Fig. 5). Soft lateral and opisthogastric integument finely plicate, bearing nine pairs of short smooth setae, 11–20 long, most of which slightly thickened basally; soft cuticle with five pairs of poroids (4 *ivo*, *idR3*), and one subcircular platelet bearing two pore-like structures (putatively a gland pore, and an associated poroid), near posterolateral margin of peritrematal-metapodal shield.

**Figure 3.**
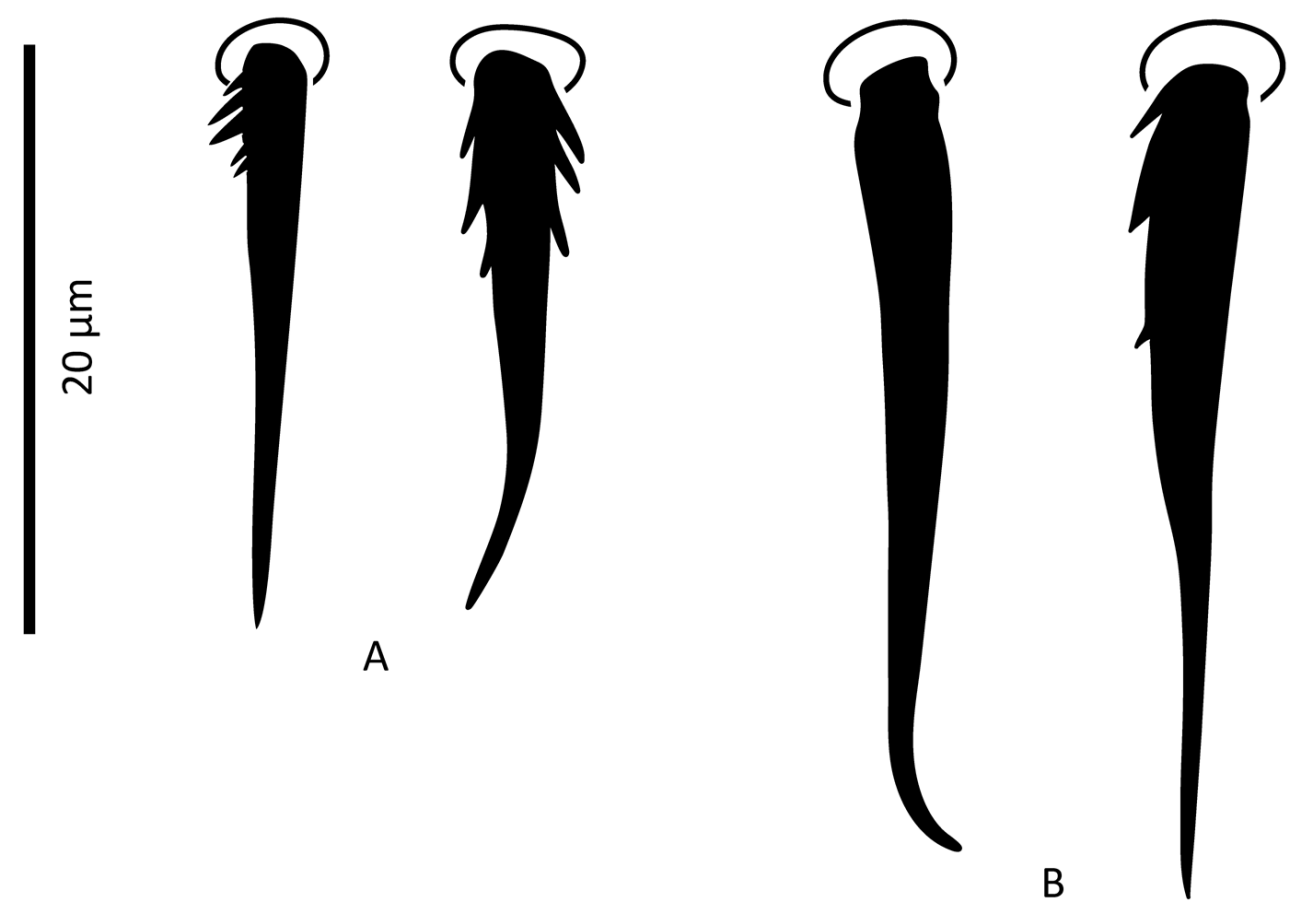
*Zygoseius papaver* sp. n., female, **A** seta *J5* **B** seta *J4*.

**Figure 4.**
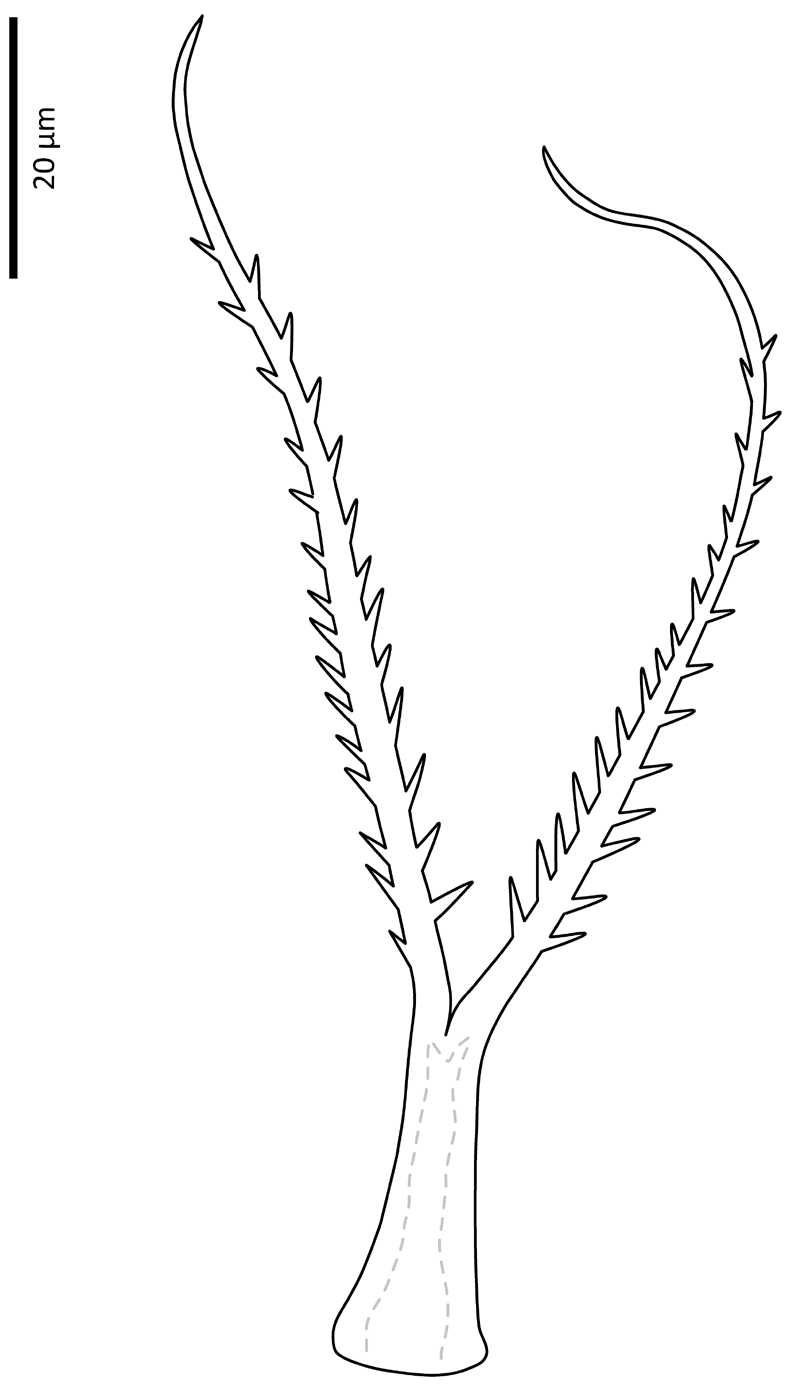
*Zygoseius papaver* sp. n., female, tritosternum.

**Figure 5.**
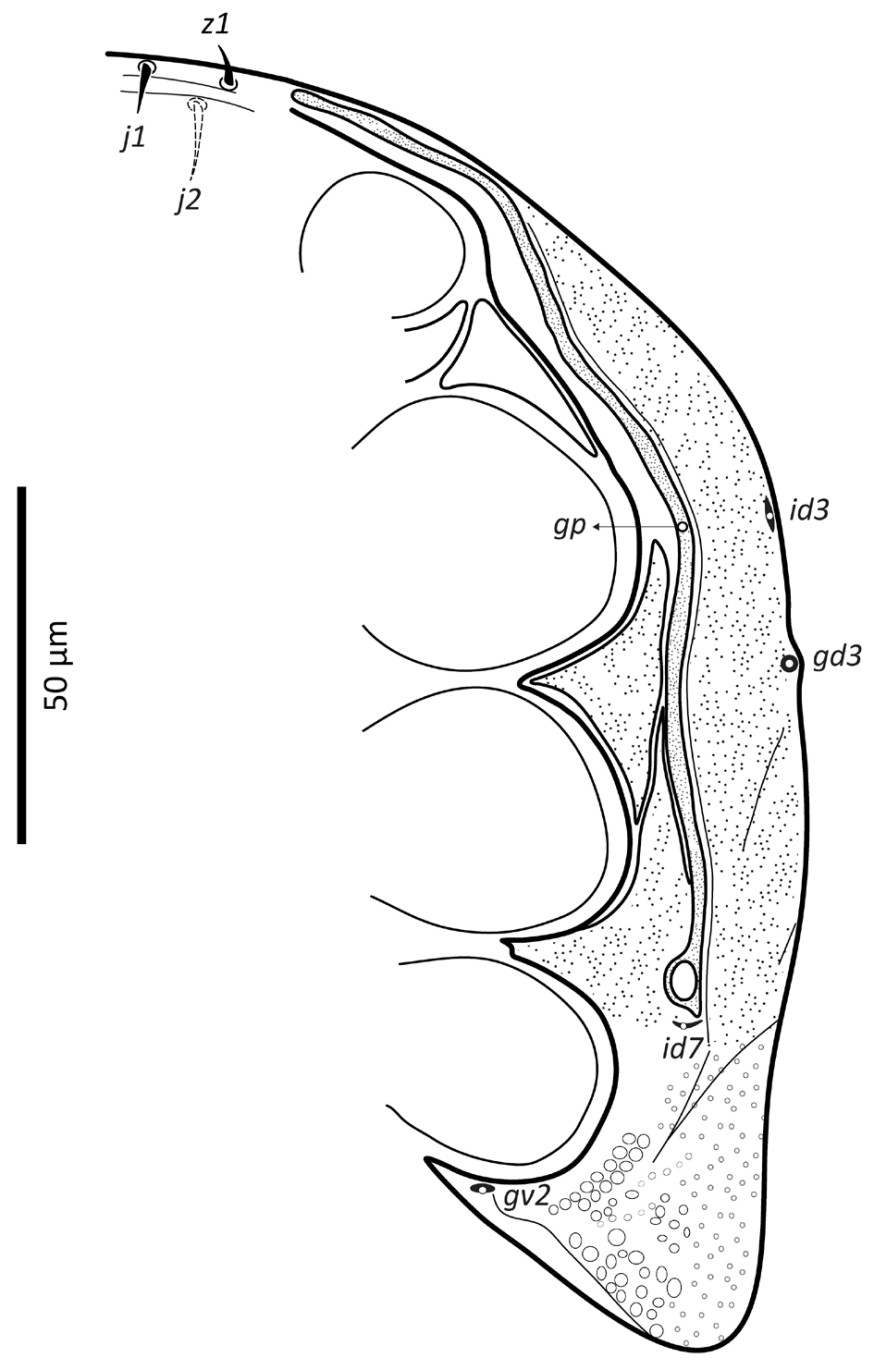
*Zygoseius papaver* sp. n., female, peritrematal shield.

**Figure 6.**
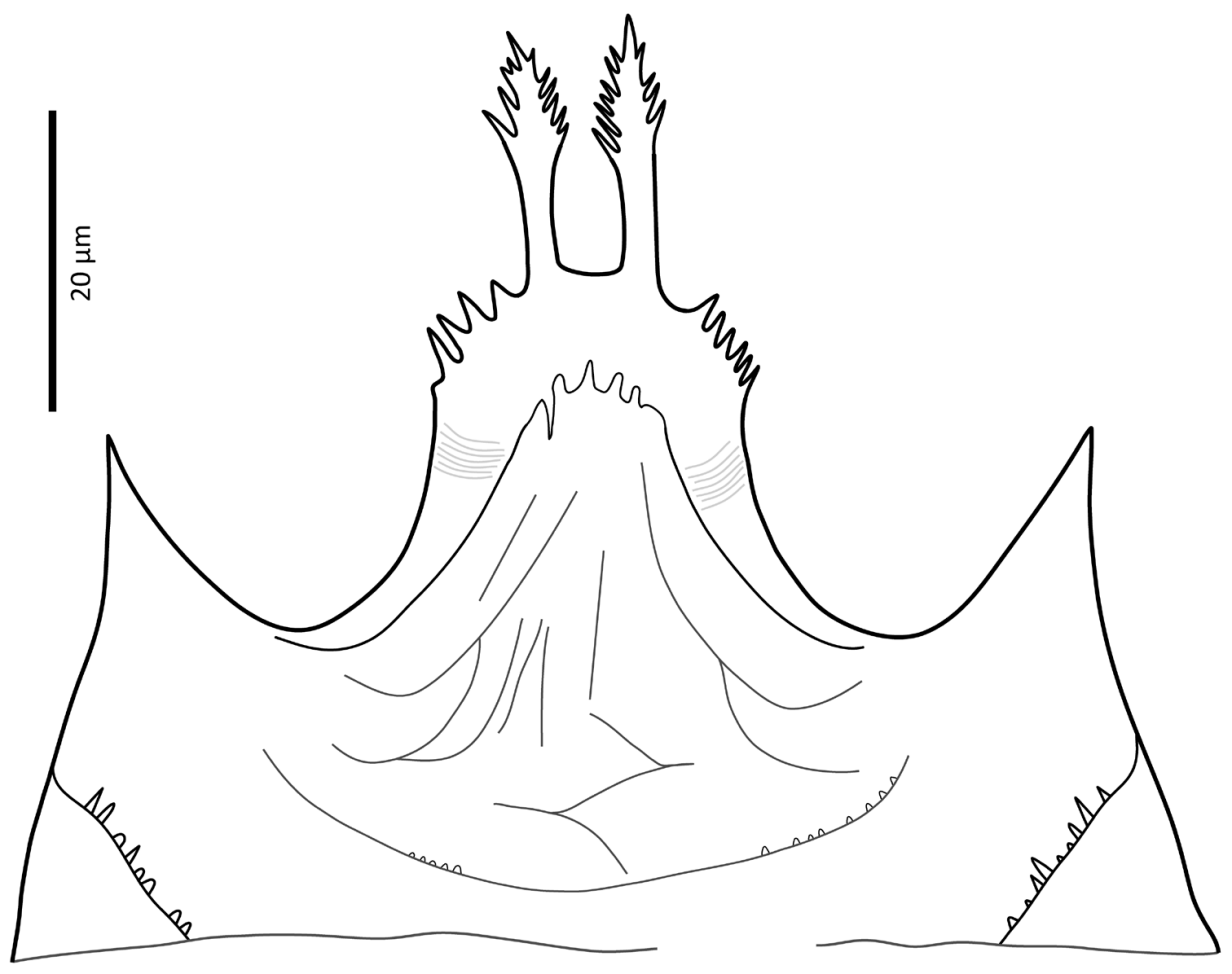
*Zygoseius papaver* sp. n., female, epistome.

**Figure 7.**
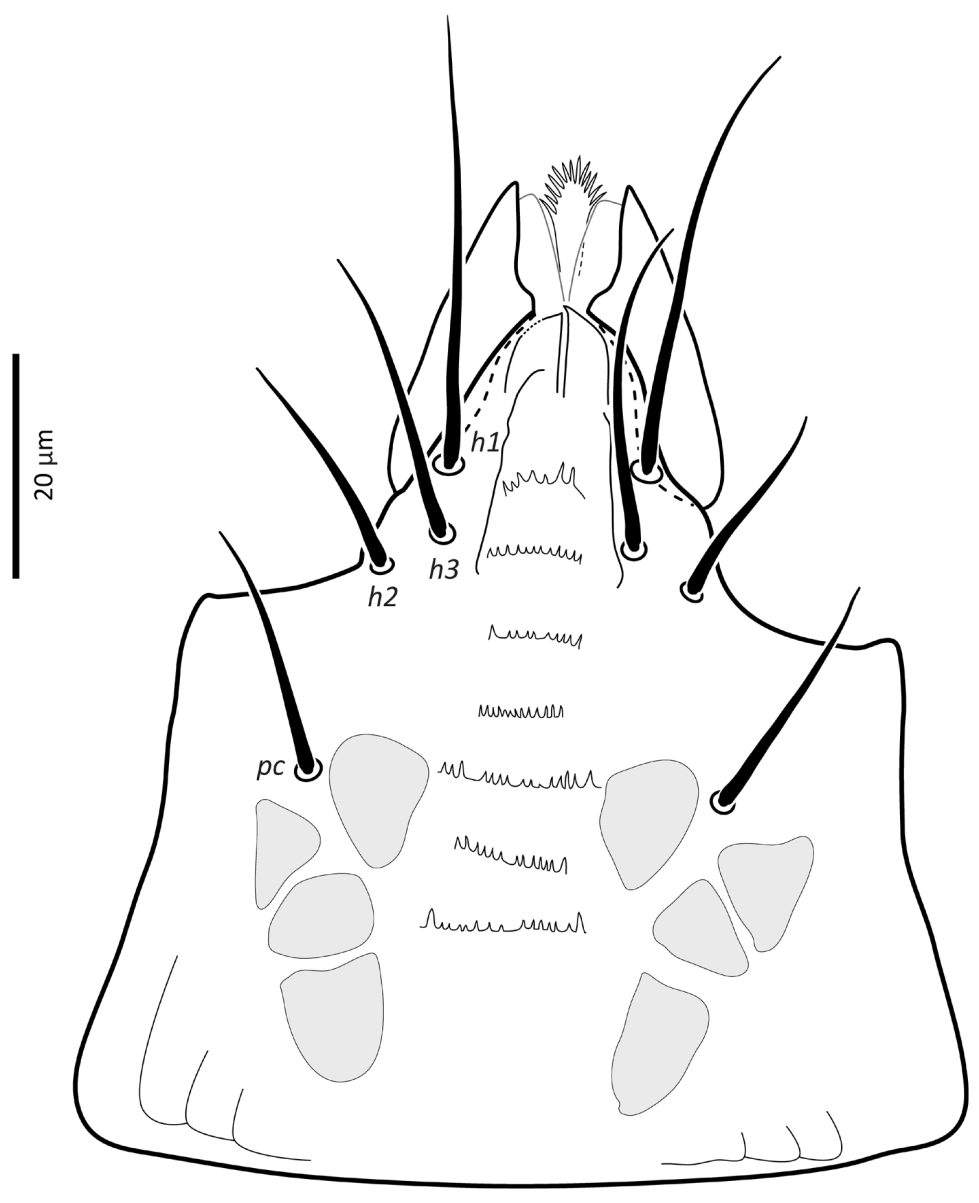
*Zygoseius papaver* sp. n., female, subcapitulum.

**Figure 8.**
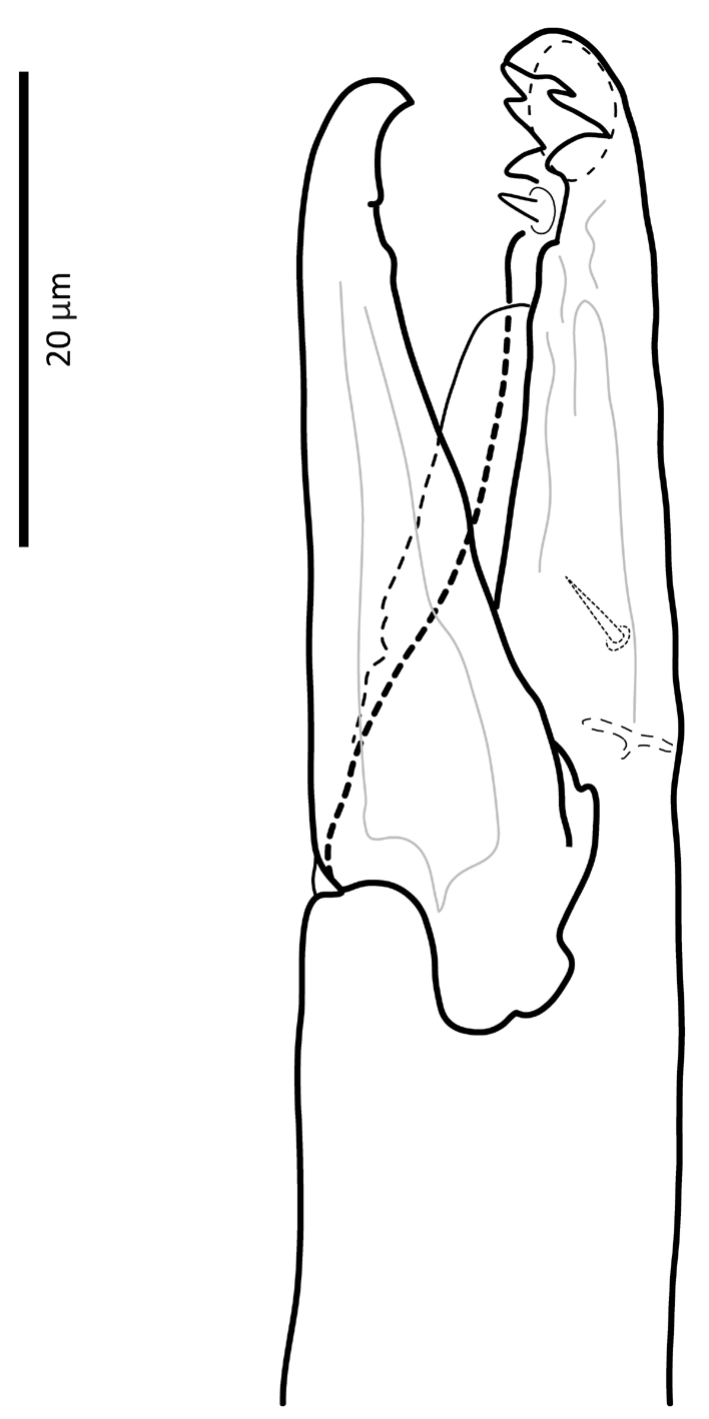
*Zygoseius papaver* sp. n., female, chelicera, ventro-paraxial view.

**Figure 9.**
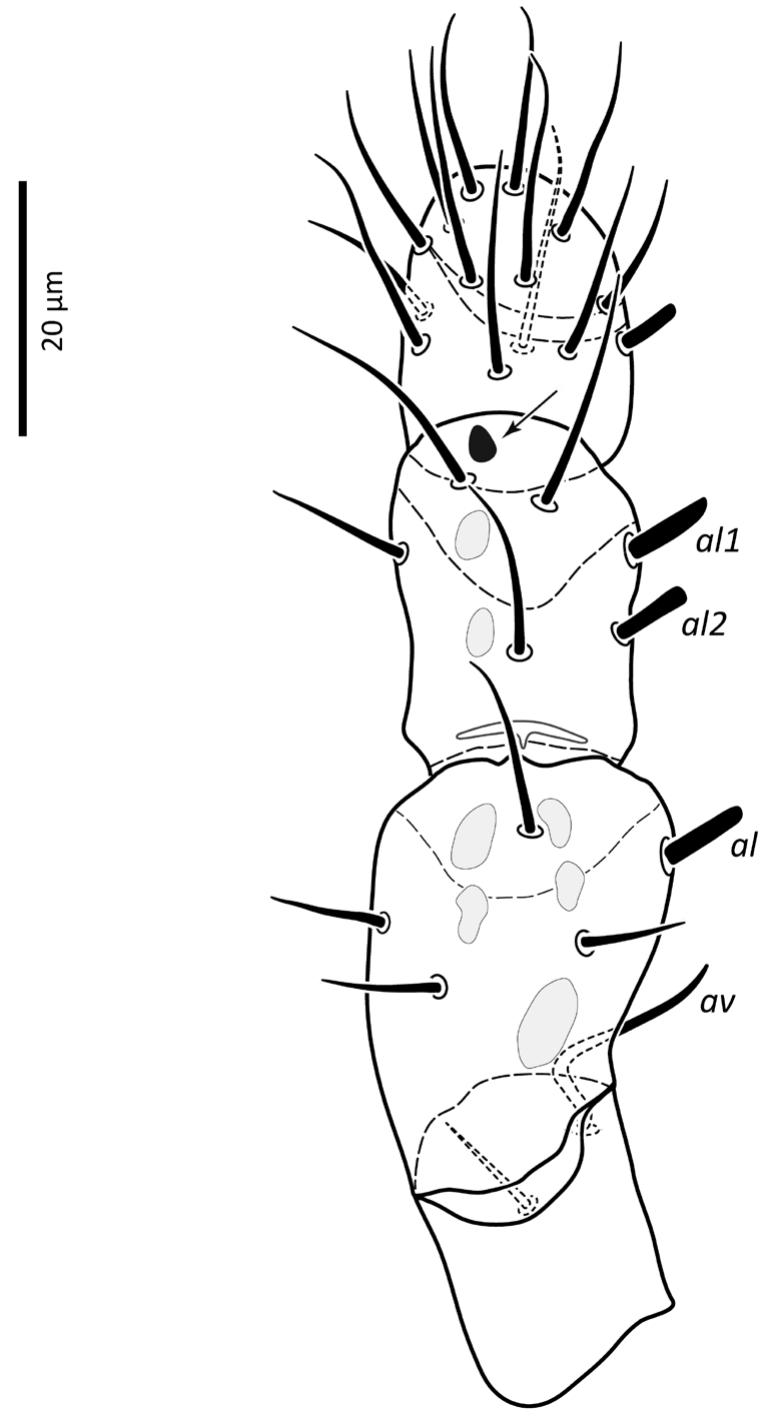
*Zygoseius papaver* sp. n., female, palp, excluding tarsus, dorsal view.

*Gnathosoma*. Epistome (Fig. 6) bifurcate, with two long (12–20) and relatively thick projections, forming a U-shape at their bases (separated by 4–7); distal halves of projections deeply serrated on both inner and outer margins, margins proximally smooth; basal margins coarsely serrated laterally. Posteromedian ridge with denticles in lateral portions; larger denticles or tubercles on posterolateral ridges. Corniculi (Fig. 7) 28–31 long, horn-like. Internal malae (Fig. 7) with a pair of smooth lobes, apically blunt, membranous, almost reaching apex of corniculi; labrum longer than internal malae, fimbriate distally. Hypostomal and capitular setae (Fig. 7) smooth, needle-like, *h1* (39–45)>*h3* (24–31)>*pc* (17–24)≈*h2* (17–21). Deutosternum (Fig. 7) with seven transverse rows of denticles; rows broad, variable in width, 5^th^ and 7^th^, or 5–7^th^ rows usually broader, anteriormost (first) row with larger denticles; numbers of teeth in rows from anterior row (1^st^) to posterior row (7^th^), respectively: 7–9, 12, 10–12, 13–14, 14–15, 13–15, 13–15. Chelicera (Fig. 8) with movable digit with two subapical, inconspicuous teeth; fixed digit with two subapical teeth followed by a short, relatively thick pilus dentilis; dorsal cheliceral seta short, setiform; first cheliceral segment 34–55 long, second 103–110 (17–28 wide), fixed digit 29–33, movable digit 34–40. Palp (Fig. 9) 101–107 long, with dorsal surfaces of genu and especially femur with some sigillae; trochanter 11–14 long, femur 31–37, genu 27–30, tibia 19–22; apotele 3-tined. Palp chaetotaxy: from trochanter–tibia 2-5-6-14 setae; trochanter 0 0/1 0/1 0, femur 1 2/0 1/0 1, genu 2 2/0 1/0 1 and tibia as in Fig. 9; all palp setae smooth, tapered; *av* (*v2*, *sensu* Evans 1963b) on trochanter strongly bent inwards (Fig. 27); *al* on femur, *al1*–*2* on genu and one of *al* setae on tibia short and spatulate; genu with stout spur dorsodistally (see arrow, Fig. 9).

**Figures 10–13.**
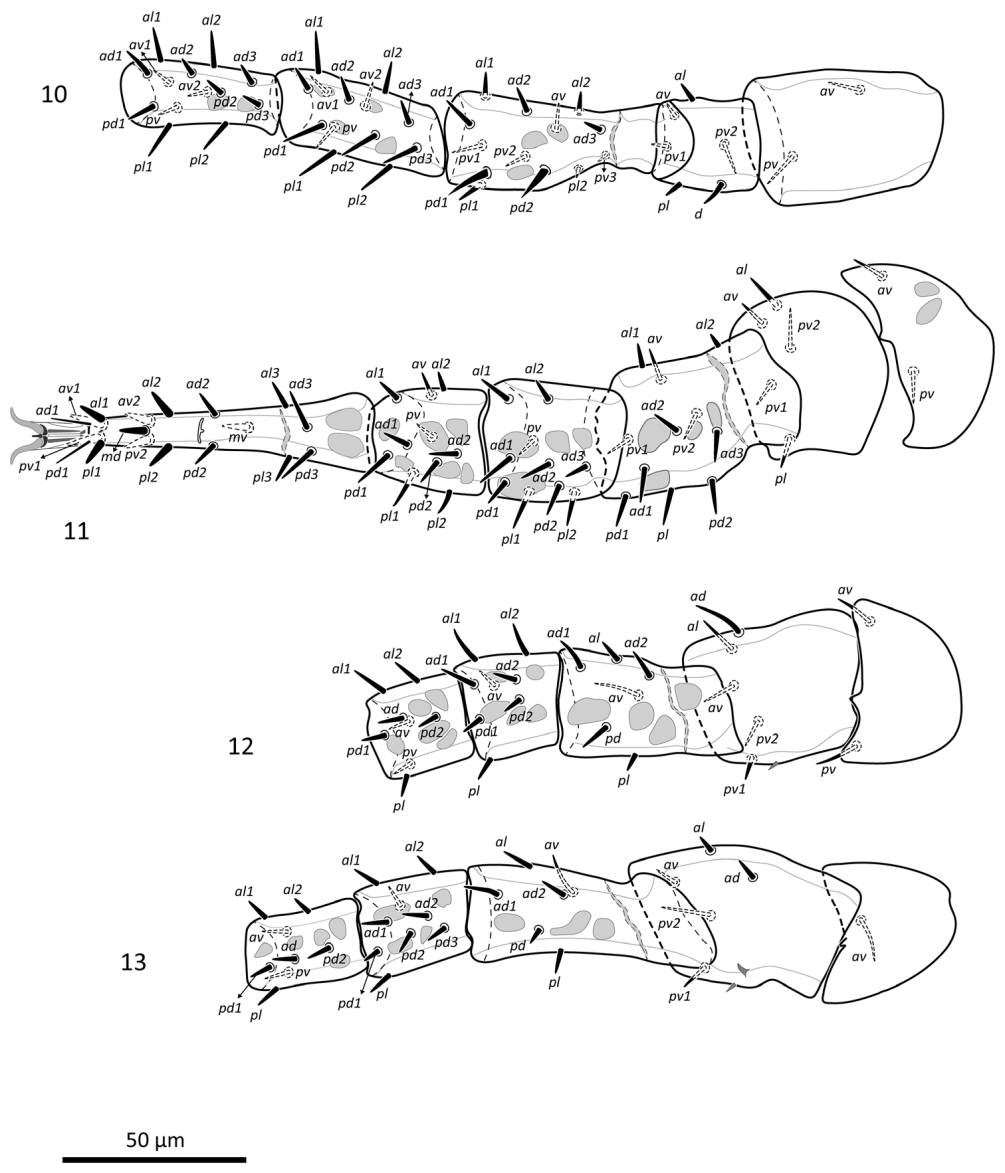
*Zygoseius papaver* sp. n., female, legs I–IV, dorsal view.

*Legs* (Figs 10–13). Lengths of legs: I 265–305, II 253–279, III 234–250, IV 271–300. Lengths of femora: I 56–64, II 42–58, III 45–53, IV 58–68; genua: I 45–49, II 36–41, III 25–30, IV 27–32; tibiae: I 40–46, II 29–36, III 27–29, IV 30–36; tarsi: I 57–65, II 73–85, III 67–73, IV 82–95; ambulacra: I 20–23, II 20–24, III 19–22, IV 22–25. Chaetotaxy of leg segments I–IV normal for *Zygoseius* (*sensu* Halliday 1997) except for genu II and genu III: coxae 2-2-2-1, or I–III (0 0/1 0/1 0), IV (0 0/1 0/0 0); trochanters 6-5-5-5, or I (1 0/1 1/2 1), II (1 0/1 0/2 1), III–IV (1 1/1 0/2 0); femora 13-11-6-6, or I (2 3/1 2/3 2), II (2 3/1 2/2 1), III–IV (1 2/1 1/0 1); genua 13-10-8-9, or I (2 3/2 3/1 2), II (2 3/0 2/1 2), III (2 2/1 2/0 1), IV (2 2/1 3/0 1); tibiae 13-10-8-8, or I (2 3/2 3/1 2 in 10 females or 2 4/2 3/1 2 in one of the 11 females), II (2 2/1 2/1 2), III–IV (2 1/1 2/1 1); tarsi II–IV 18-18-18, all as 3 3/2 3/2 3 + *md* and *mv*. All setae on legs I–IV simple, relatively short and tapered, except: femur I with *pd1*–*2* thickened (lengths: *pd1* 12–13, *pd2* 10–11); tarsi II–IV with apical setae *al1*, *av1*, *pv1*, *pl1* and subapical setae *av2*, *pv2*, *md* and *mv* short, spur-like. Trochanter III with small cuticular spur posterolaterally, and trochanter IV with two cuticular spurs, posterolaterally and posterodorsally. Sigillae on ventral surfaces of coxae I–IV and trochanters I–II, and dorsal surfaces of femora, genua and tibiae I–IV, and basitarsi II–IV. All ambulacra with a pair of well-developed hooked claws. Pulvilli not discerned.

**Plate 1.**
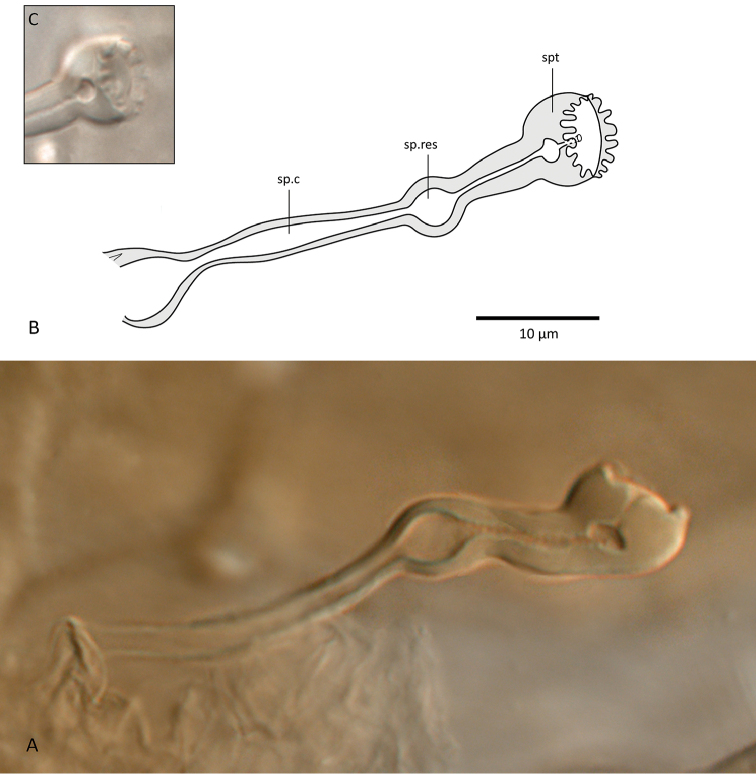
*Zygoseius papaver* sp. n., female, **A, B** spermathecal apparatus in two different females. Abbreviations: sp.c.= spermatic canal, sp.res.= sperm reservoir, spt.= spermatheca **C** spermatheca.

*Spermathecal apparatus* (Plate 1). Spermatheca (Plate 1C) globular, large (diameter 8–11), connected to a short, thick-walled duct (5–10 long), followed by a small ring-like sperm reservoir (diameter 5–6), and a narrow and long spermatic canal (16–24 long), sometimes widened basally (as in Plate 1B).

*Male* (n = 1). *Dorsal idiosoma* (Fig. 30). Dorsal shield oval, 338 long, 252 wide (length/width ratio: 1.34), completely covering idiosoma. Shield ornamentation and chaetotaxy similar to those of female, except reticulation in central region of idiosoma between setae *j6*–*j6* to *J2*–*J2* more distinct.

*Ventral idiosoma* (Fig. 31). Tritosternum as in female, 14 long, 11 wide proximally, 6 wide apically; laciniae 76 long. Gonopore diameter 20, discernible part of duct 50 long. Holoventral shield 271 long, 217 wide (length/width ratio: 1.25), reticulate nearly throughout except between setae *st5*–*JV1*, cells punctate inside and along margins; ventral region weakly lineate and punctate between setae *JV1* and *JV2*, with more distinct punctae laterally and especially posteriorly. Holoventral shield fused laterally to peritrematal, metapodal and exopodal elements, bearing 12 pairs of simple and smooth setae (five and seven pairs on sternogenital and ventrianal regions, respectively) (Table 1), and three smooth circum-anal setae; shield with nine pairs of pore-like structures (*iv1–3*, *iv5*, *gv2–3*, three pairs of *ivo*), excluding those on peritrematal-exopodal shields. Setae *JV1*–*2* longer than other ventral setae, including *JV3*–*5*, *ZV1*–*3* (Table 1). Peritreme 178 long. Soft lateral and opisthogastric integument with 6–7 pairs of short setae, 7–15 long, slightly thickened basally, and two or three pairs of pore-like structures. Anal opening subtriangular, 22 long and 19 wide. Other features of ventral idiosoma as in female.

*Gnathosoma*. Epistome as in female, with two projections, 19 long, distance between bases of projections 5. Corniculi (26 long) and deutosternum as in female. Lengths of hypostomal setae: *h1* 39, *h2* 14, *h3* 24, *pc* 19. Chelicera and spermatodactyl not available for study (broken off specimen). Palp 98 long, similar to that of female; trochanter 13 long, femur 40, genu 22, tibia about 21; palp setae and chaetotaxy as in female.

**Figure 14.**
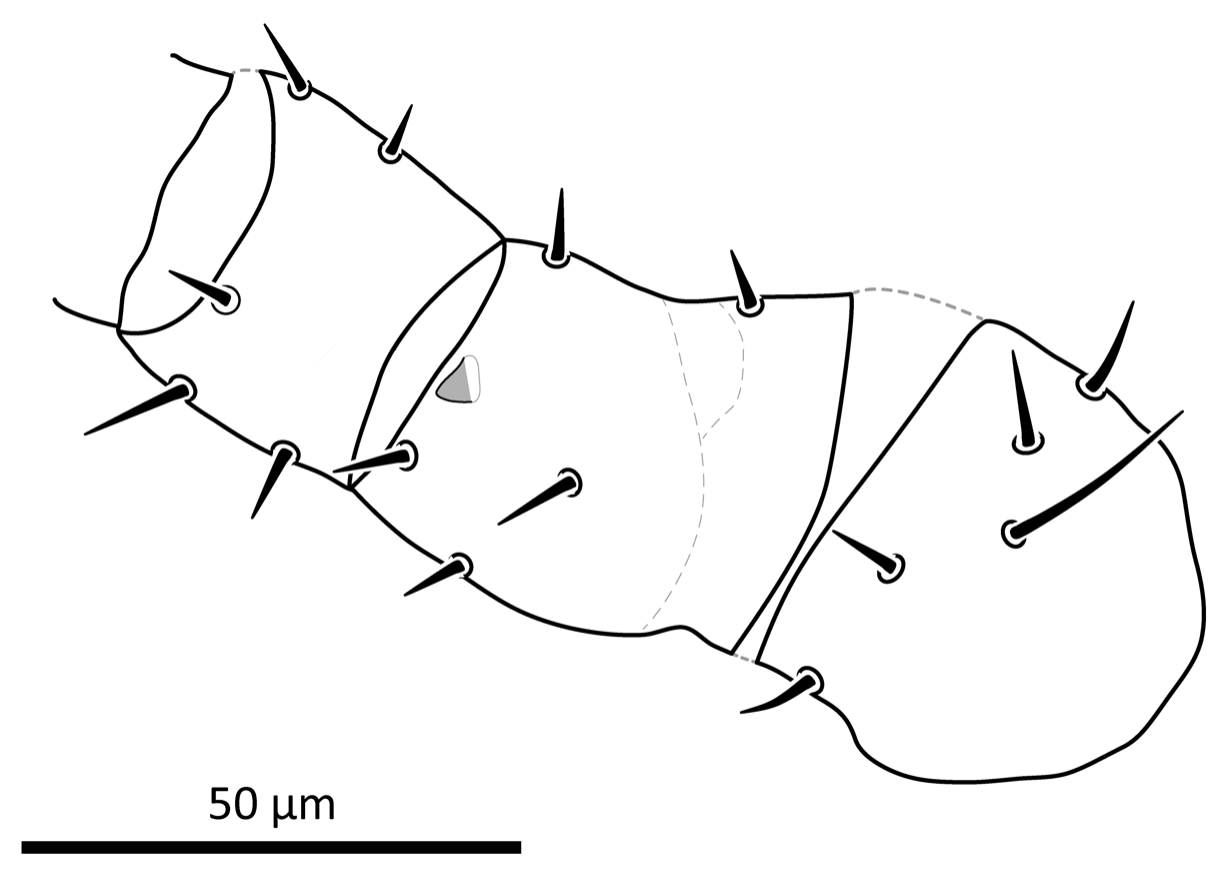
*Zygoseius papaver* sp. n., male, trochanter-genu II, ventral view.

*Legs*. Lengths of legs: I 288, II 239, III 231, IV 288. Lengths of femora: I 61, II 44, III 55, IV 60; genua: I 45, II 37, III 26, IV 30; tibiae: I 44, II 32, III 25, IV 31; tarsi: I 61, II 71, III 68, IV 87, ambulacra: I 18, II 20, III 19, IV 24. Chaetotaxy of legs I–IV similar to that of female, except that the femur II has one conical spine-like projection ventrodistally (Fig. 14). Setae *pd1*–*2* on femur I thickened as in female, *pd1* 14–15, *pd2* 10–12. Sigillae locations similar to those of female.

#### Immature stages.

Unknown.

#### Material examined.

Holotype: Female. Mexico, Chiapas State, Volcan Tzontehuitz, 9000 ft. (= 2743.2 m. a.s.l.), 12 miles NE of San Cristóbal de Las Casas, from moss on log, 19 May 1969, coll. J. M. Campbell. Paratypes: 15 females, 1 male, same data as holotype. The holotype and 12 paratypes (females and male) are deposited at the Canadian National Collection of Insects, Arachnids and Nematodes (CNC) at the Agriculture and Agri-Food Canada, Ottawa, Canada, and four female paratypes are deposited at the Acarology Collection of the Department of Entomology
(ACDE), College of Agriculture and Natural Resources, Science and Research Branch, Islamic Azad University, Tehran, Iran.

#### Etymology.

The specific name refers to the shape of the spermatheca of the new species, which resembles the capsule of opium (*Papaver somniferum* L., 1753). It is considered as a noun in apposition.

#### Remarks.

The spermathecal apparatus of *Zygoseius papaver* sp. n. is distinct from that of any other *Zygoseius* species for which it was described: the spermetheca is globular and larger than any other sclerotized part of the apparatus, and ends in a flower-like pattern. The new species can also be distinguished by its long *J1*–*2* setae relative to the distance between *J1* and *J2* setae (ratio setal length/distance = 0.90 ± 0.06 st.dev., range 0.75–1.0). Based on their illustrations, a few species described from South America have long *J1*–*2* setae relative to the distance between them, such as *Zygoseius alveolaris* Karg, 1998 and *Zygoseius triramuli* Karg & Schorlemmer, 2009 (Karg 1998, Karg and Schorlemmer 2009), but these have a different arrangement of setae of the *j*–*J* series, including the presence of *J3*.

The epistome of *Zygoseius papaver* sp. n. is unique among described species, with relatively short but thick projections that are conspicuously barbed apically. The epistome of *Zygoseius laticuspidis* Karg, 1998 is similar; however, it is even more swollen apically, and is slightly denticulate on the basal margin in-between the projections. *Zygoseius laticuspidis* also has *J5* setae inserted mesad of *Z5* (note, however, that the relative position of *J5* and *Z5* can vary, depending on how flattened is the dorsal shield on the slide). The new species can further be distinguished from *Zygoseius laticuspidis* by its shorter dorsal setae (all are <30 long; most are 30–60 long in *Zygoseius laticuspidis*), *J4* setae separated by 1.4–1.9× the distance between *J1* setae (*J4*–*J4* distance over twice that between *J1*–*J1* in *Zygoseius laticuspidis*), and by the presence of nine pairs of setae on the opisthogastric soft cuticle (six pairs in *Zygoseius laticuspidis*). Other *Zygoseius* species can be distinguished from *Zygoseius papaver* sp. n. by some of the same characters mentioned above, as well as by (1) its epistome; (2) the length and width (and their ratios) of the dorsal, sternal and ventrianal shields; (3) relative length of dorsal setae, especially *Z5*; (4) the ornamentation of the dorsal and sternal shields; and (5) long *JV1–2* setae, 1.5–2× as long as other pre-anal setae on the ventrianal shield, and as long as about 2/3 of distance between *JV1* and *JV2. Zygoseiusampullus* Halliday, 1997 and *Zygoseius foramenis* Karg, 1998 also have longer *JV1*–*2* setae but clearly differ by their epistomes, and by shorter *J1–2* setae and a ventrianal shield as long as wide. In the key to species of Karg and Schorlemmer (2009), *Zygoseius papaver* sp. n. would reach couplet 3 (12), and can be distinguished from species in (3) and (12) by the characters mentioned above.

Another distinguishing feature of *Zygoseius papaver* sp. n. is the distinctly serrated lateral margins of the dorsal shield. This also characterizes *Zygoseius ovatus* Karg, 1998. The margins of the dorsal shield of other species may appear somewhat serrated (e.g. *Zygoseius ampullus*, *Zygoseius metoecus* Halliday, 1997 and *Zygoseius separatoporus* Karg, 1998), although the serration matches with the insertion of setae in marginal positions (mostly *r* and *S* setae), whereas in the new species and at least in *Zygoseius ovatus*, most serration are independent of setal insertions. Such serrated margins of the dorsal shield are reminiscent of the dorsal shield of many Zerconidae (Ujvári 2010, 2011) and some species of *Pachyseius* Berlese (Pachylaelapidae) (Mašán 2007, Ahadiyat et al. 2016). Note that the serration of dorsal shields in zerconid and *Pachyseius* species is largely correlated, although not entirely, with the insertion of marginal setae.

*Zygoseius papaver* sp. n. also differs from other *Zygoseius* species by its reduced chaetotaxy on genu II, lacking seta *av*, and genu III, lacking seta *pv*, instead of the usual complement of two ventral setae, including both *av* and *pv* as noted in the genus diagnosis of Halliday (1997). His diagnosis was based on four species (*Zygoseius furciger*, *Zygoseius ampullus*, *Zygoseius metoecus*, *Zygoseius sarcinulus*), so we can predict that other described (with unstudied leg chaetotaxy) and undescribed species have such genual chaetotaxy. However, because at least another species of *Zygoseius*, newly described herein (see below), sometimes lacks *pv* on genu III, we can suspect that other species also lacks such seta. Members of other non-parasitic dermanyssine families lack both of these setae (e.g. Phytoseiidae; Evans 1963a), or lacks either *av* on genu II (some *Pseudolaelaps* species, Pseudolaelapidae; Mašán 2014) or more commonly *pv* on genu III (e.g. some Eviphididae, Pachylaelapidae, Macrochelidae, Ascoidea, Blattisociidae; Evans 1963a, Lindquist and Evans 1965, Moraza and Johnston 1990, Mašán 2007, Mašán and Halliday 2010), showing plasticity of the development of those setae. Based on the studied chaetotaxy of *Zygoseius furciger* and of other dermanyssines (Evans and Till 1965, Lindquist and Evans 1965, Halliday 1997), when present in the adults, ventral setae of genua II–III appear at the deutonymphal stage. Therefore, they are theoretically not as stable as (i.e. less likely to be retained in the adult stage than) setae appearing at an earlier developmental stage (Evans 1963a, Lindquist and Evans 1965, Rowell et al. 1978).

### Zygoseius lindquisti sp. n.

Taxon classificationAnimaliaMesostigmataPachylaelapidae

http://zoobank.org/50B0C71A-5F59-4852-B39E-C9D5E78895FB

Figures 15 , 16 , 17 , 18 , 19 , 20 , 21 , 22 , 23–26 , 27 , 32 , 33 , Plate 2

#### Diagnosis.

Dorsal shield oval, densely micropunctate, with relatively distinct reticulation and lineation, except more weakly reticulated medially between setae *j4–6*. Edges of lateral parts of dorsum smooth. Dorsal setae smooth, except *J4* and *J5* with a few barbs basally; all setae less than 35 long; setae *z6*, *s6*, and all opisthonotal setae (except *J5* and *Z5*) 1.5–2× as long as other setae. Sternal shield densely micropunctate, except in the regions of setal insertions. Epigynal shield conspicuously punctate in anterior 2/3, punctae lighter posteriorly. Ventrianal shield distinctly lineate in anterior half, reticulate laterally and posteriorly; setae *JV2* slightly longer than other setae on shield. Peritrematal shield micropunctate throughout, punctae larger in poststigmatic region. Soft lateral and opisthogastric cuticle with nine pairs of setae. Epistome bifurcate, thin projections slightly converging, about twice as long as distance between their bases, sparsely serrated in apical half. Hypostomal setae *h1* about twice as long as *h2*, and subequal to *h3*. Femur I with seta *pd2* thickened. Spermathecal apparatus with a small, kidney-shaped spermatheca directly connected to a globular, large sperm reservoir, followed by a long spermatic canal with diverging walls.

#### Description

**Figure 15.**
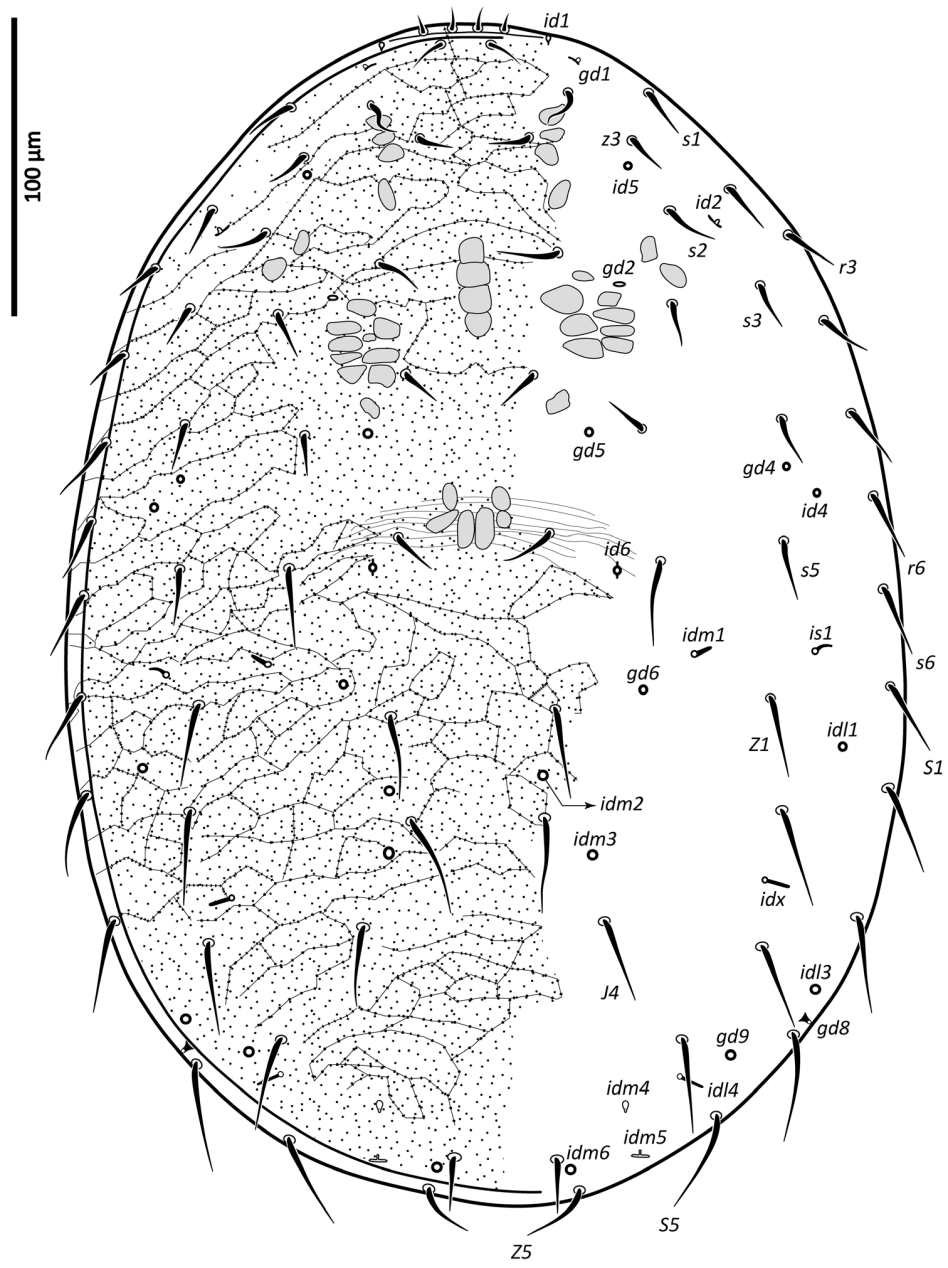
*Zygoseius lindquisti* sp. n., female, dorsal idiosoma.

*Female* (n = 2). *Dorsal idiosoma* (Figs 15, 32). Dorsal shield oval, 396–413 long, 278–283 wide (length/width ratio: 1.40–1.48), completely covering idiosoma; edges of lateral parts of dorsum smooth, with no marginal serration; shield densely micropunctate throughout, distinctly reticulate-lineate, more weakly reticulate medially, especially between setae *j4*–*j6* and posterad setae *Z3–4* and around and posterad *J5*. Dorsal shield with 37 pairs of setae, 23 and 14 pairs on podonotal and opisthonotal regions, respectively; lacking setae *J3*. Dorsal setae less than 35 long, all smooth, acuminate, slightly swollen basally, except *J4–5* finely pilose basally (Fig. 17A, B). Opisthonotal setae about twice as long as podonotal setae (Table 1). Dorsal idiosoma with 23 pairs of pore-like structures, including seven gland openings and 16 poroids.

**Figure 16.**
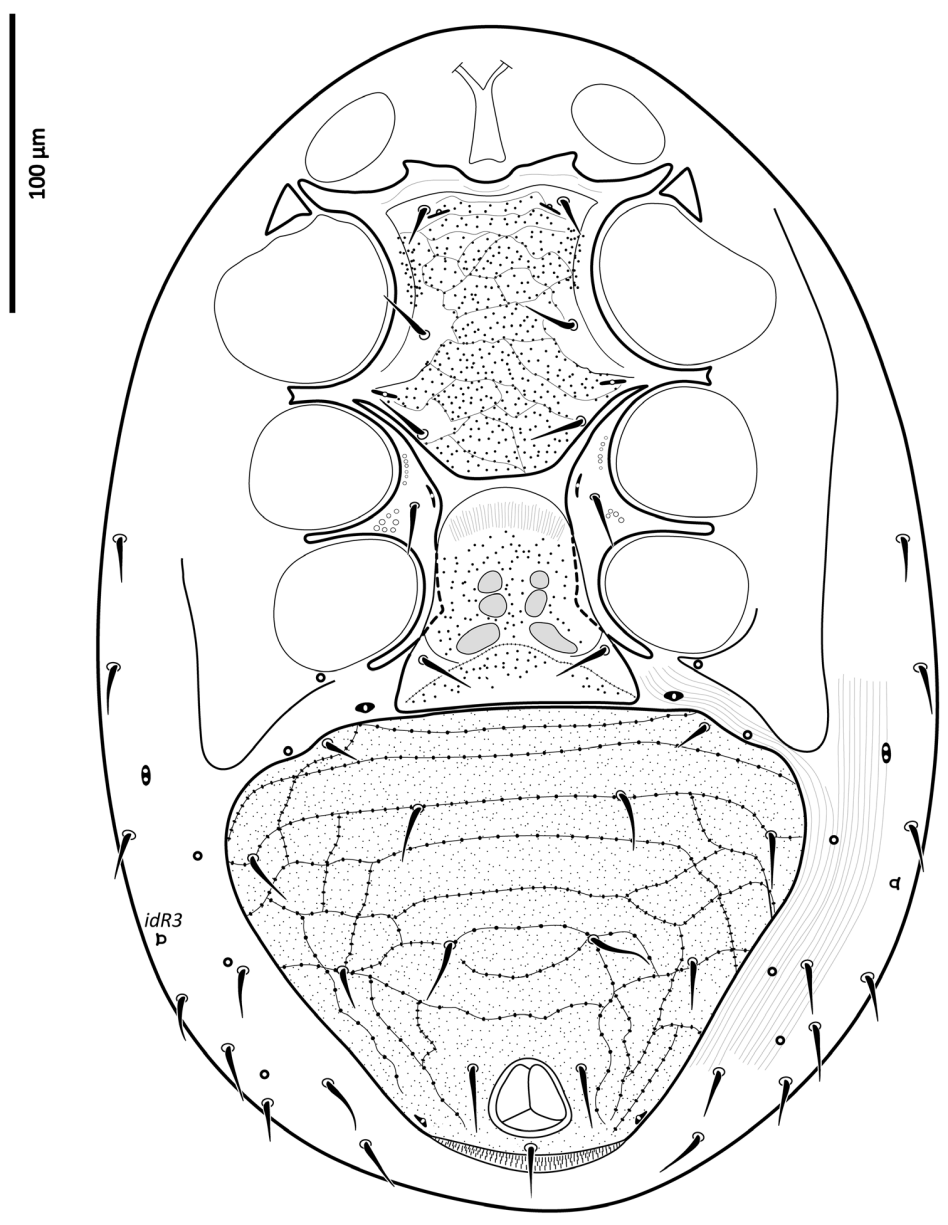
*Zygoseius lindquisti* sp. n., female, ventral idiosoma.

*Ventral idiosoma* (Figs 16, 33). Tritosternum with a trapezoidal base, 23–28 long, 12–14 wide proximally, 4–6 wide apically, and a pair of laciniae (61–64 long). Laciniae with barbs relatively short and blunt (Fig. 18). Sternal shield 98–102 long, 66–71 wide (length/width ratio: 1.44–1.48), bearing two pairs of poroids and three pairs of smooth, subequal setae *st1–3* (Table 1); shield anterolateral arms long, contiguous to subtriangular exopodal plate between coxae I and II; anterior margin with distinct median notch and two subtriangular projections; posterior margin truncate; shield densely micropunctate throughout, except smooth around sternal setae. Complex of metasternal and endopodal elements arc-shaped, mostly smooth, punctate in restricted areas, bearing simple setae *st4* and poroids *iv3*. Epigynal shield trapezoidal, 85–87 long, 22–24 long from *st5* to posterior margin, 81–84 wide (length/width ratio: 1.03–1.07), conspicuously punctate in anterior 2/3, punctae lighter posteriorly; shield with transverse convex line passing behind setae *st5*; anterior hyaline portion rounded, indistinct; shield closely abutting ventrianal shield; three pairs of suboval to subcircular sigillae medially, posterior ones larger, oval. Setae *st5* smooth, inserted near shield lateral margins. Poroids *iv5* near posterolateral margins of epigynal shield. Ventrianal shield subpentagonal, broad, 153–154 long, 189–196 wide (length/width ratio: 0.79–0.81), with straight anterior margin; distinctly lineate in anterior half, reticulate laterally and posteriorly; cells micropunctate inside and along cell margins; shield bearing five pairs of pre-anal and three circum-anal setae, all smooth; setae *JV2* slightly longer than other setae; other setae subequal, except *ZV1* shorter (Table 1); para-anal setae inserted at level of anterior margin of anal opening; gland openings *gv3* on posterolateral margins of shield at level of posterior margin of anus; cribrum well-developed, 2–3 rows of spicules, extending along posterior shield margin between *gv3* openings; anal opening 25–26 long, 21–22 wide, subtriangular to subcircular, located in posterior fifth or fourth of shield. Peritreme 191–198 long, densely covered with aciculae, extending anteriorly near seta *z1*, with one gland pore (*gp*) at mid-level of coxa II. Peritrematal shield wide, fused to exopodal, parapodal and metapodal elements, extending well behind posterior level of coxae IV; shield micropunctate, with larger punctae in poststigmatic region, with four pore-like structures (*id3*, *gd3*, *id7*, *gv2*). Exopodal element between coxae II–III fused with other exopodal-peritrematal elements (Fig. 19). Soft lateral and opisthogastric integument plicate, bearing nine pairs of setae, 15–30 long, slightly thickened basally, marginal setae as the longest. Soft cuticle with five pairs of poroids, including four *ivo*, *idR3*, and an oval platelet bearing two pore-like structures, at level of posterior margin of peritrematal shield.

**Figure 17.**
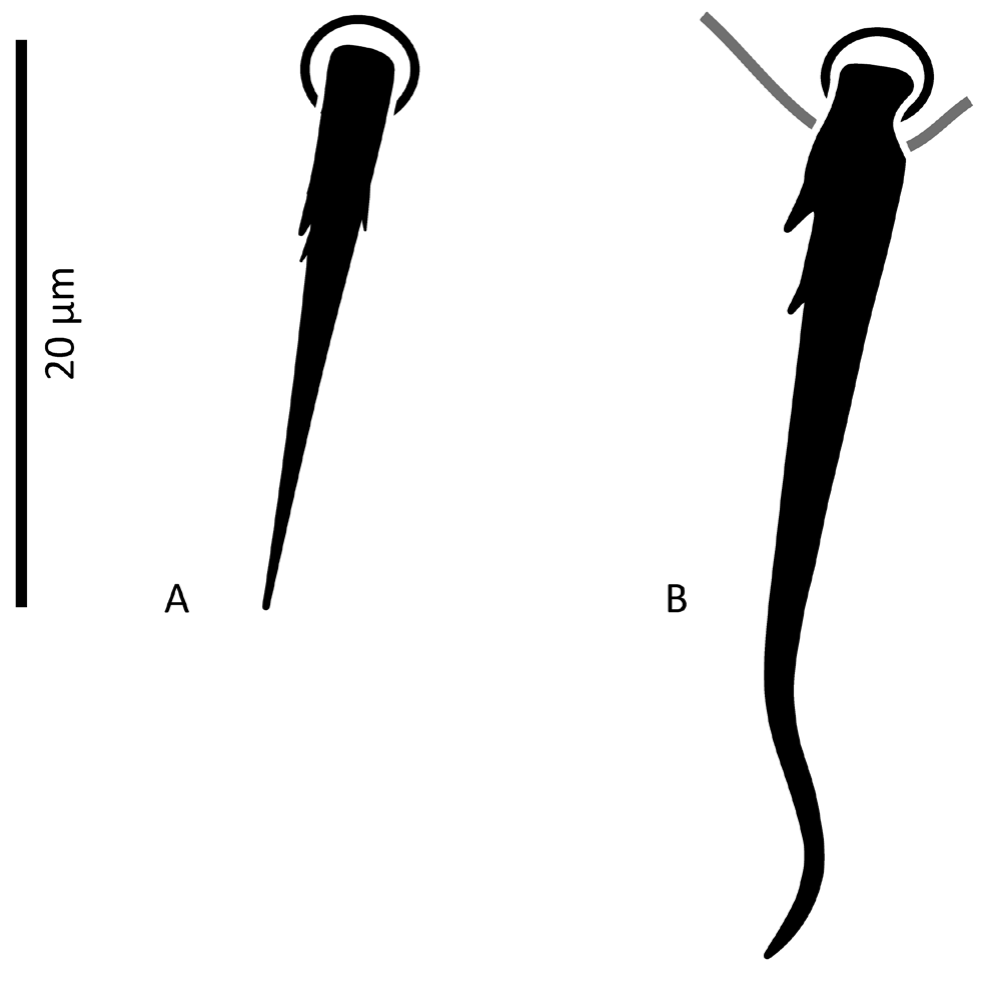
*Zygoseius lindquisti* sp. n., female, **A** seta *J5* **B** seta *J4*.

**Figure 18.**
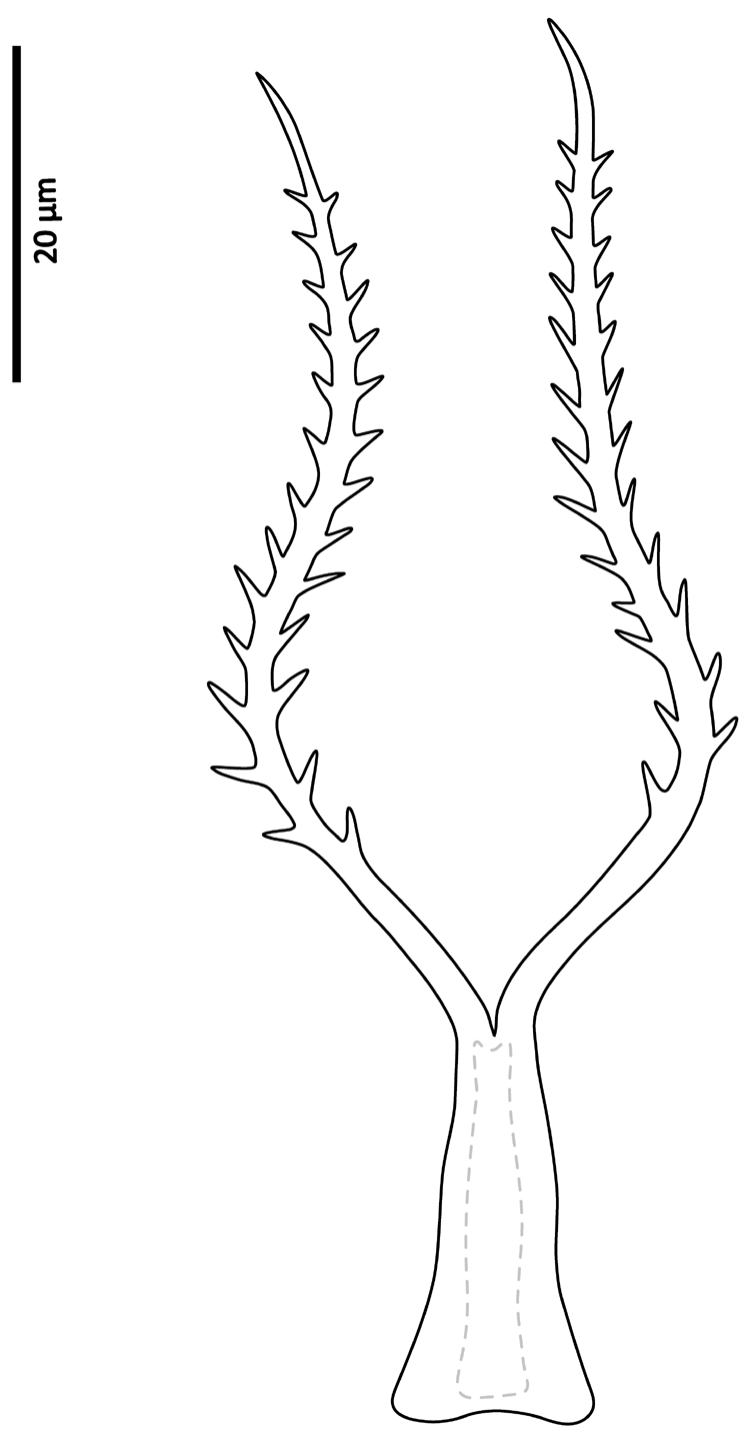
*Zygoseius lindquisti* sp. n., female, tritosternum.

**Figure 19.**
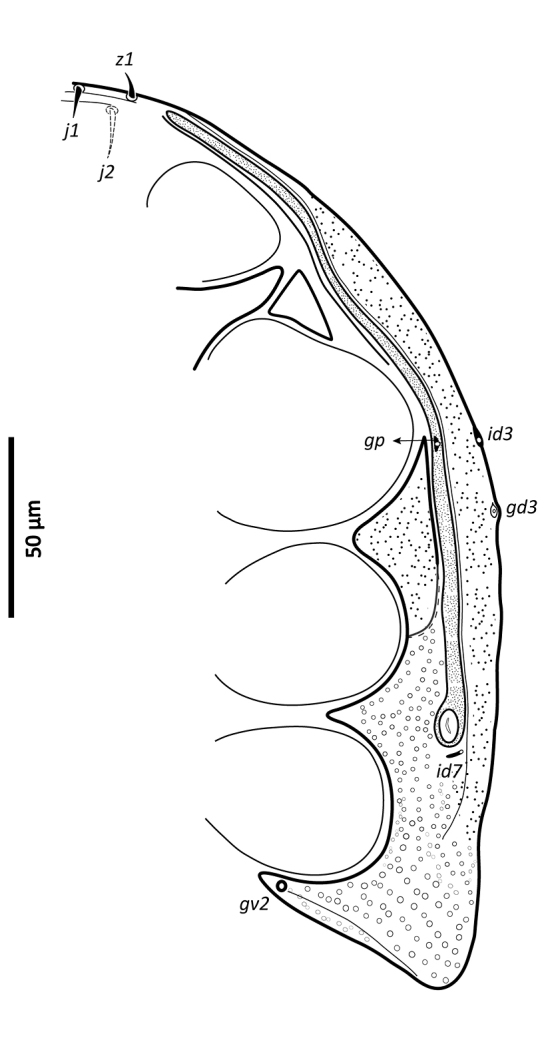
*Zygoseius lindquisti* sp. n., female, peritrematal shield.

**Figure 20.**
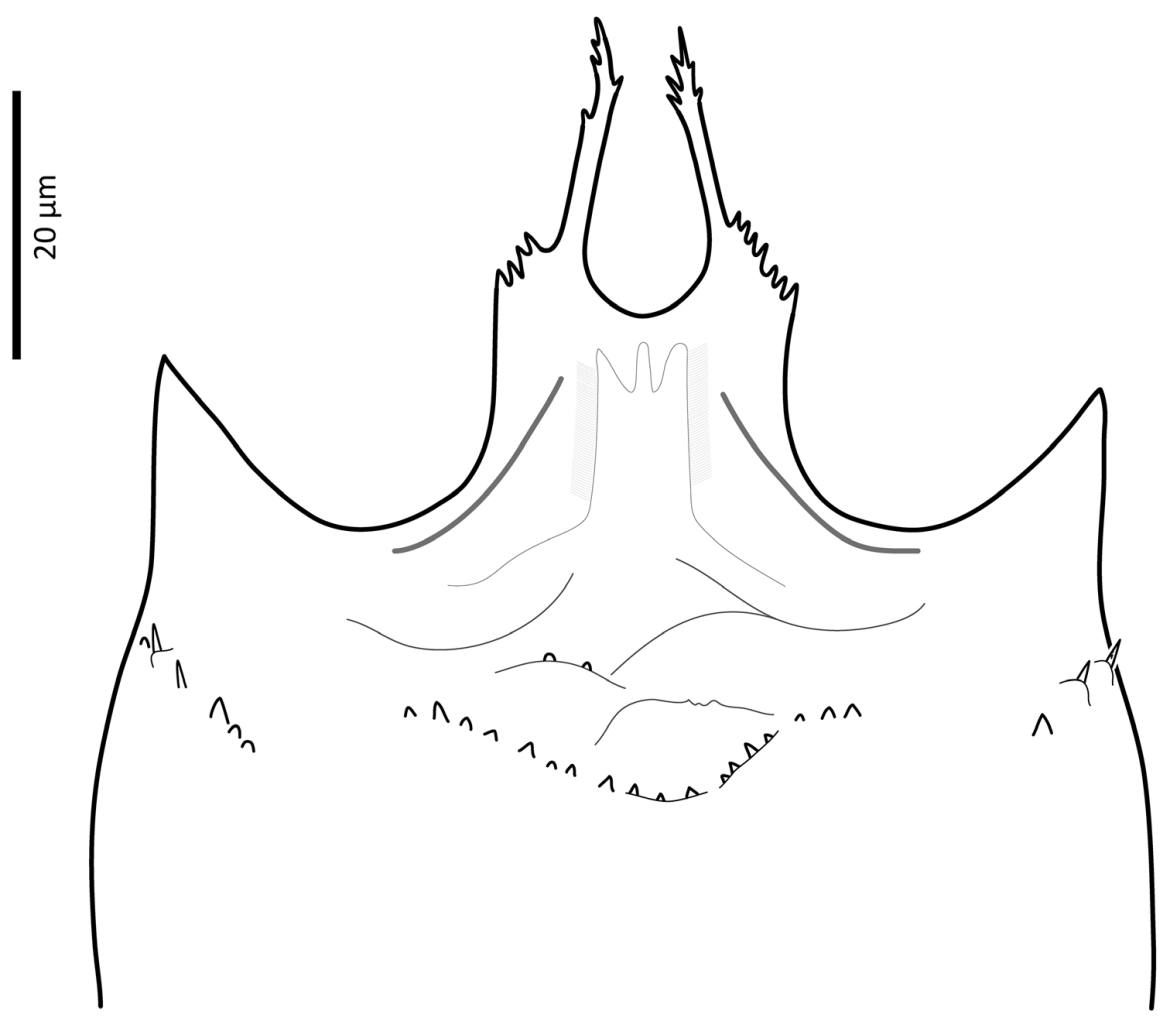
*Zygoseius lindquisti* sp. n., female, epistome.

**Figure 21.**
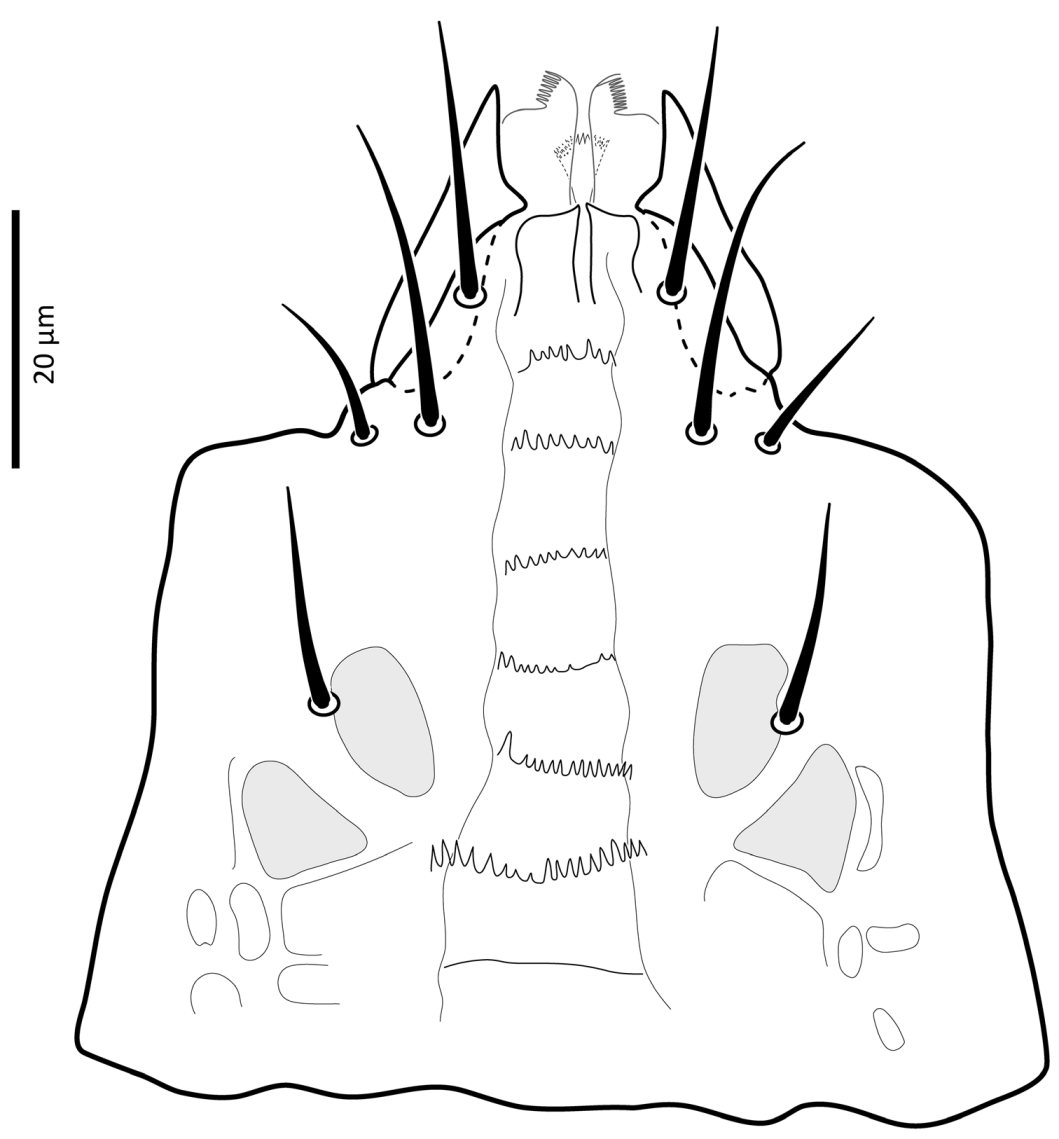
*Zygoseius lindquisti* sp. n., female, subcapitulum.

**Figure 22.**
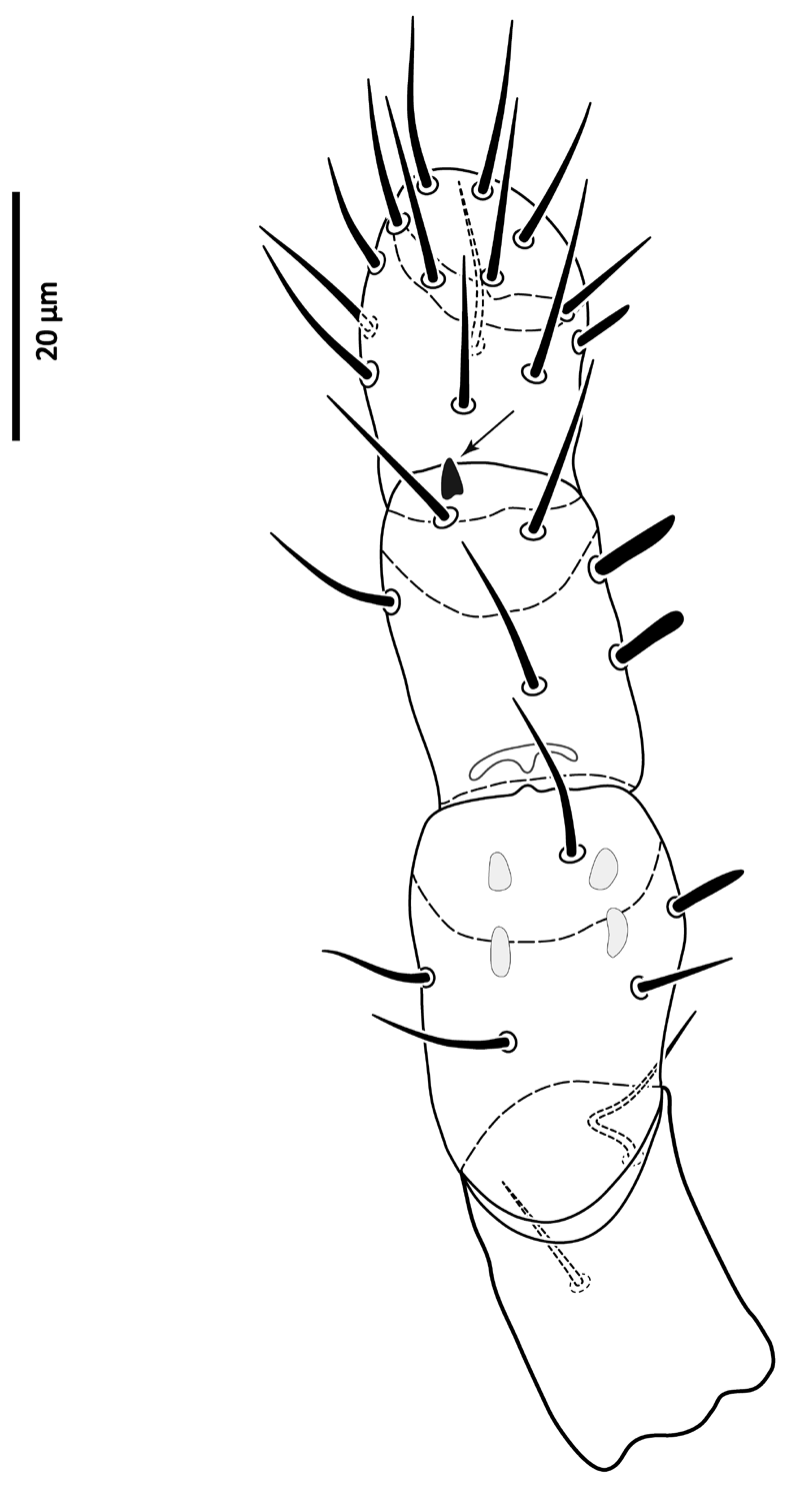
*Zygoseius lindquisti* sp. n., female, palp, excluding tarsus, dorsal view.

*Gnathosoma*. Epistome (Fig. 20) bifurcate, with two slender projections (16–20 long), forming a U shape at their bases (separated by 8–10), slightly converging; distal halves of projections sparsely serrated on inner margin (in one specimen) or both inner and outer margins (in other specimen), margins proximally smooth; basal margin finely serrated laterally; a transverse series of blunt to sharp tubercles posteromedially, and fewer series laterally. Corniculi (Fig. 21) short, 24–26, horn-like. Internal malae (Fig. 21) finely developed, reaching slightly beyond corniculi; anterolateral margins fimbriate, inner margins smooth; labrum fine, shorter than internal malae, finely fimbriate distally. Hypostomal and capitular setae (Fig. 21) smooth, needle-like, *h3* (21–about 28) and *h1* (21–25)>*pc* (about 13–17)>*h2* (8–9). Deutosternum (Fig. 21) with 6–7 transverse rows of denticles, followed posteriorly by a smooth ridge; posteriormost row of denticles widest; two anteriormost (1^st^ and 2^nd^) and posterior-most (5^th^ and/or 6^th^) rows with larger denticles; numbers of denticles from anterior to posterior rows: 8–10, ~ 9, 10–11, ~ 10–11, 12–14, 15–18. Cheliceral teeth not clearly discernable (digits oriented dorsoventrally); first cheliceral segment 35–44 long, second segment and fixed digit unclear; movable digit 27–29; width of second segment 17–21. Palp (Fig. 22) 105–113 long, dorsal surfaces of femur and genu with some sigillae; trochanter 13–18 long, femur 34–36, genu 27–29, tibia 23–26; apotele 3-tined. Palp chaetotaxy: from trochanter–tibia 2-5-6-14 setae; trochanter 0 0/1 0/1 0, femur 1 2/0 1/0 1, genu 2 2/0 1/0 1; tibia as in Fig. 22. All palpal setae smooth, tapered; *av* (*v2*, *sensu* Evans 1963b) on trochanter strongly bent inwards (Fig. 27); *al* on femur, *al1*–*2* on genu and one of *al* setae on tibia short and spatulate; genu with stout spur dorsodistally (see arrow, Fig. 22).

**Figures 23–26.**
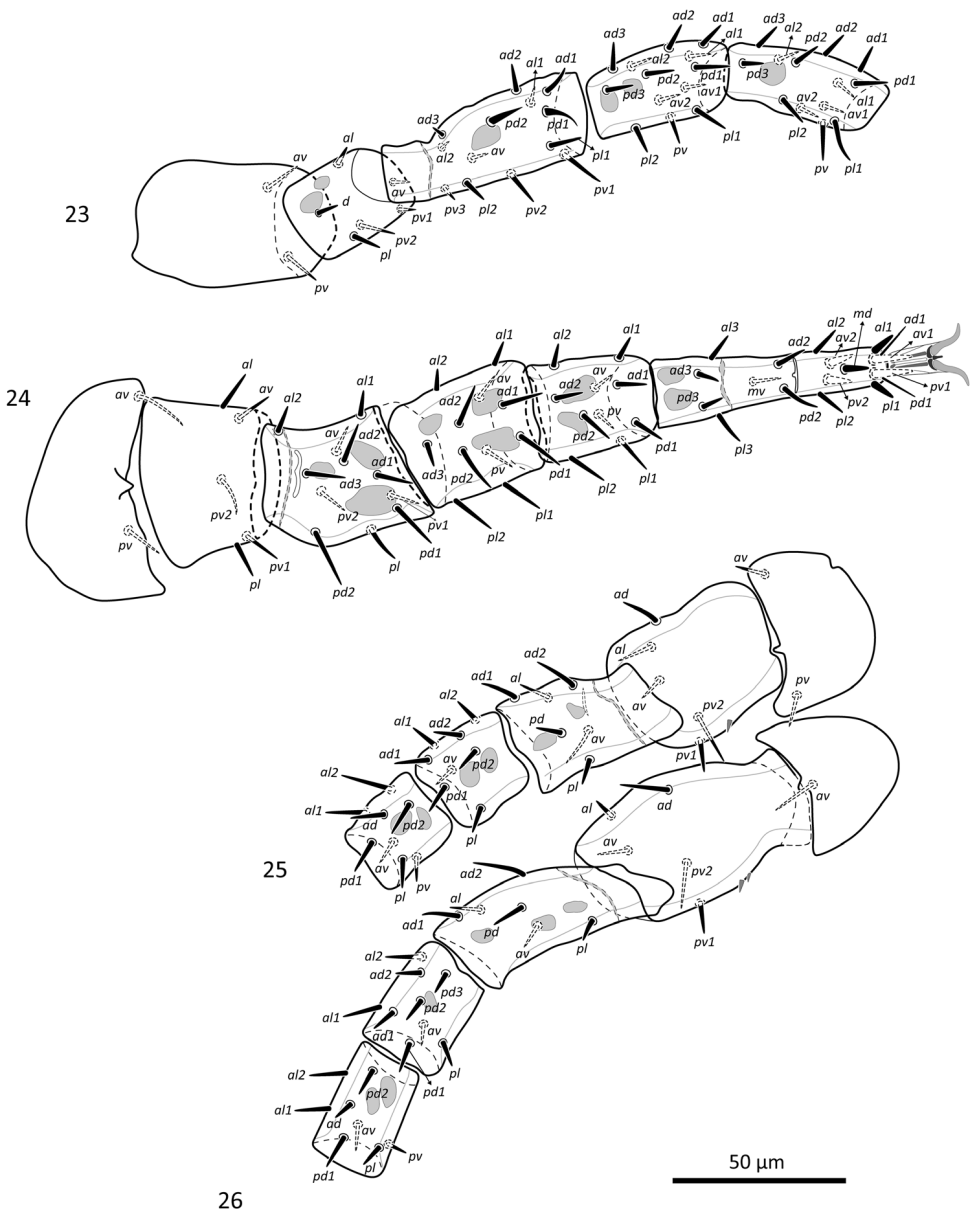
*Zygoseius lindquisti* sp. n., female, legs I–IV, dorsal view.

*Legs* (Figs 23–26). Lengths of legs: I 295–307, II 257–261, III 233–241, IV 307–309. Lengths of femora: I 60–63, II 49–52, III 48–53, IV 64–66; genua: I 44–45, II 42–44, III 24–27, IV 31–34; tibiae: I 42–45, II 33–36, III 28–29, IV 36–38; tarsi: I 66–72, II 68–73, III 63–65, IV 88–91; ambulacra: I 21–25, II 21–22, III 19–20, IV 20–22. Chaetotaxy of leg segments I–IV normal for *Zygoseius* (*sensu* Halliday 1997): coxae 2-2-2-1, or I–III (0 0/1 0/1 0), IV (0 0/1 0/0 0); trochanters 6-5-5-5, or I (1 0/1 1/2 1); II (1 0/1 0/2 1), III–IV (1 1/1 0/2 0); femora 13-11-6-6, or I (2 3/1 2/3 2), II (2 3/1 2/2 1), III–IV (1 2/1 1/0 1); genua 13-11-8 or 9-9, or I (2 3/2 3/1 2), II (2 3/1 2/1 2), III (2 2/1 2/0 1 in one specimen, or 2 2/1 2/1 1 in another specimen), IV (2 2/1 3/0 1); tibiae 13-10-8-8, or I (2 3/2 3/1 2), II (2 2/1 2/1 2), III–IV (2 1/1 2/1 1); tarsi II–IV 18-18-18, all as 3 3/2 3/2 3 + *md* and *mv*. All setae on legs I–IV simple, relatively short and tapered, except: femur I with *pd1*–*2* thickened, *pd2* thicker (lengths: *pd1* 10–12, *pd2* 11–12); tarsi II–III with apical setae *al1*, *av1*, *pv1*, *pl1* and subapical setae *av2*, *pv2* and *md* short, spur-like; tarsus IV with setae *al1*, *av1*, *pv1*, *pl1* and *md* short, spur-like; tarsi II–IV with *mv* longer and slightly slender. Trochanter III with small cuticular spur posterolaterally, and trochanter IV with two cuticular spur posterolaterally. Ventral surfaces of coxae II–IV and trochanters I–II, anterolateral surface of trochanter IV, and dorsal surfaces of femora and tibiae I–IV, genua and basitarsi II–IV with some sigillae. All ambulacra with a pair of well-developed hooked claws. Pulvilli not discerned.

**Plate 2.**
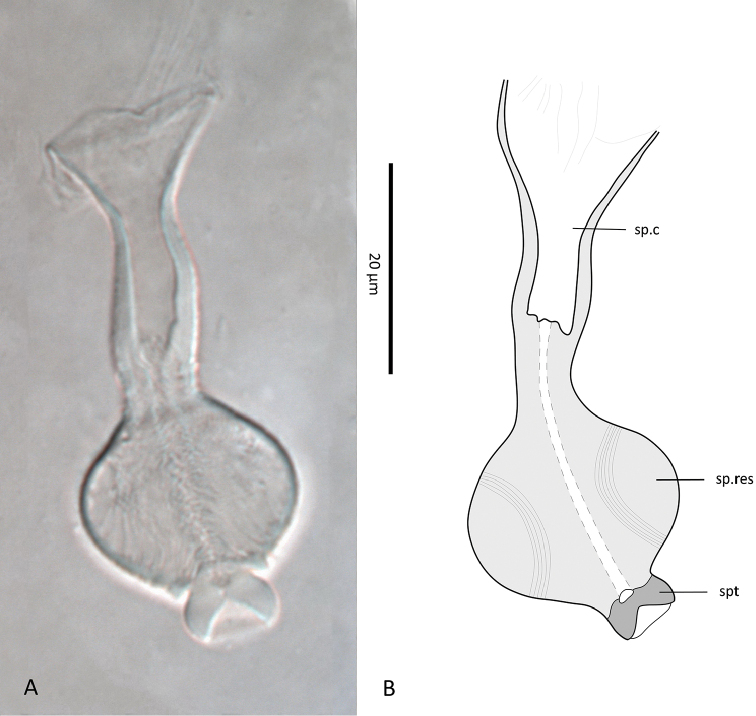
*Zygoseius lindquisti* sp. n., female, **A, B** spermathecal apparatus in two different females (Abbreviations as mentioned in Plate 1).

*Spermathecal apparatus* (Plate 2). Spermatheca small, 6–8 wide, somewhat kidney-shaped, with no stalk, directly connected to a globular, large sperm reservoir (diameter 17–21), followed by a long spermatic canal (27–34 long). Sperm reservoir presenting a narrow central duct; spermatic canal with distinct walls, diverging basally.

#### Male and immature stages.

Unknown.

#### Material examined.

Holotype: Female. Mexico, Chiapas State, 6 miles NE of San Cristóbal de Las Casas, from flood debris in creek, 15 May 1969, coll. Evert E. Lindquist. Paratype: Female, same data as holotype. The holotype and paratype are deposited at the Canadian National Collection of Insects, Arachnids and Nematodes (CNC), Agriculture and Agri-Food Canada, Ottawa, Canada.

#### Etymology.

The species is named in honor of Evert E. Lindquist, for his invaluable endeavors on the systematics of Mesostigmata over the years. The specimens of this new species were collected by him.

#### Remarks.

The dorsal seta of trochanter I in *Zygoseius papaver* and *Zygoseius lindquisti* is inserted in a posterior position. We herein call this seta *d* (Figs 10, 23), although in the chaetotactic formula, we indicated it as posterodorsal, given its clear posterior position, as in Halliday (1997). Evans (1963a, fig. 1i) indicated ‘*ad*’ for this dorsal seta, as illustrated for *Pergamasus* (Parasitidae). In the text, however, he called it ‘*d*’, for *Pergamasus* and for other gamasines. We have examined adult specimens of other *Zygoseius* spp., as well as of *Pachylaelaps* (Pachylaelapidae), *Gaeolaelaps* (Laelapidae), *Asca* (Ascidae), *Proctolaelaps* (Melicharidae), *Parasitus* and *Pergamasus* (Parasitidae), and the dorsal seta of trochanter I was usually inserted in a slightly to moderately posterior position, and rarely on the mediodorsal line or in a (slightly) anterior position.

In his diagnosis of the genus *Zygoseius*, Halliday (1997) indicated one *pv* and one *pl* setae on trochanter IV, whereas Evans (1963a) indicated two *pv* and no *pl* (as we did, herein). Indeed, *pv1* is inserted much more posteriorly than *pv2* (although not necessarily posterolaterally), and this situation is similar to that of *pv1*–*2* of trochanters II–III (Evans 1963a; Figs 11–13, 24–26).

In addition to poroid *idR3*, between setae *R3* and *R4*, the soft opisthogastric cuticle has a sclerotized complex of two pore-like structures, posterolaterad the peritrematal-metapodal shield. These structures may be two openings of the same underlying gland complex; alternatively, they may be a gland opening and an associated poroid (note that both of these structures are sometimes visible in lateral view when the soft cuticle is folded, instead of the normal ventral view). It is unclear whether this gland opening is homologous to the one (*gp*) typically found in the poststigmatic region of peritrematal shields in many Mesostigmata (e.g. Lindquist and Moraza 2016). This double pore-like structure also occurs in *Zygoseius papaver* sp. n., as well as in *Zygoseius ampullus* and *Zygoseius metoecus* (Halliday 1997), and *Zygoseius sarcinulus* (AA, personal observations).

*Zygoseius lindquisti* sp. n. shares certain morphological features with *Zygoseius incisus* Karg, 1998 and *Zygoseius margaritatus* Karg & Schorlemmer, 2009, including: (1) an epistome with two thin projections, about twice as long as distance between their bases, sparsely serrated, mostly in apical half; (2) the ratio *J4* setae inserted well farther apart from each other than *J1* setae (ratio of distance *J4*–*J4*/*J1*–*J1*= 1.42–1.57 in *Zygoseius lindquisti* sp. n.); (3) *J1*–*2* setae slightly shorter than distance between insertions of *J1* and *J2* (length *J1*–*2* setae/*J1*–*2* distance= 0.8–0.9 in *Zygoseius lindquisti* sp. n.); (4) ventrianal shield with short setae, including *JV1*–*2*; (5) the length of seta *Z5* (20–26 in *Zygoseius lindquisti* sp. n.). It also has a spermathecal apparatus similar to *Zygoseius margaritatus*, although the latter has a more elongate, egg-shaped spermatic reservoir followed by a spermatic canal more constricted distally. The spermathecal apparatus of *Zygoseius incisus* is distinct, with a narrow elongate spermatic canal. The species *Zygoseius lindquisti* sp. n. can further be distinguished from the two species by (1) the dense micropunctation on its dorsal, sternal and genital shields, and its ventrianal shield lineate anteriorly and reticulate laterally and posteriorly; (2) its relatively broad dorsal shield (396–413 long, 278–283 wide; vs 430 long, 260 wide in *Zygoseius incisus*, 336–392 long, 231–256 wide in *Zygoseius margaritatus*); (3) its relatively wide ventrianal shield (153–154 long, 189–196 wide; vs. 160 long, 170 wide in *Zygoseius incisus*, 140 long, 182 wide in *Zygoseius margaritatus*); (4) many longer setae in the opisthonotal region (e.g. *J1*, *J4*, *S5*).

The new species also has a spermathecal apparatus similar to *Zygoseius furciger*. Based on the two females examined, however, *Zygoseius lindquisti* sp. n. has a sperm reservoir globular with enlarged spermatic canal throughout, whereas the sperm reservoir of *Zygoseius furciger* ranges from globular to oval with spermatic canal constricted distally (in proximity to sperm reservoir). The detailed description of Halliday (1997) allows to easily distinguish the new species from *Zygoseius furciger*, by (1) its sternal shield faintly lineate and densely micropunctate (reticulate and with punctae along cell margins in *Zygoseius furciger*); (2) smaller dorsal shield (396–413 long; vs 418–518 in *Zygoseius furciger*); (3) some setae in opisthonotal region slightly longer (e.g. *J1*, *J4*); (4) hypostomal setae *h1* and *h3* subequal in length (*h3* about 1.5× as long as *h1* in Halliday, 1997); (5) deutosternum with 6–7 rows of denticles (eight rows in *Zygoseius furciger*).

## Discussion

The record of a “*Zygoseius* sp.” by Palacios-Vargas (1983) probably represents from the first mention of the genus in Mexico. Among the now 15 described species, 12 are found in South America, including one (*Zygoseius furciger*) that is also found elsewhere (USA, Africa, Israel); two (described herein) occur in Mexico, and one (*Zygoseius sarcinulus*) is widespread in Australia.

Some morphological characters are of particular interest for the diagnosis of *Zygoseius* species and possibly also for classifying them into species groups. Perhaps the most useful character to distinguish *Zygoseius* species is the spermatheca itself varying in size relative to the rest of the apparatus, and the sperm reservoir varying in shape, ranging from oval to globular (Halliday 1997, Karg 1998). More detailed studies of the spermathecal apparatus will probably help further the systematics of *Zygoseius*, analogously as to its use for other Mesostigmata, such as the Phytoseiidae (Chant and McMurtry 1994, Beard 2001) and Pachylaelapidae (Mašán 2007).

The dorsal idiosomal chaetotaxy is moderately useful, with some setae varying markedly in position between species, such as *J5* relative to *Z5*, and with the atypical presence of seta *J3* in some species (in *Zygoseius triramuli* and *Zygoseius alveolaris*; Karg 1998). Although Halliday (1997) stressed the difficulty in using shield ornamentation (e.g. sternal shield) for species discrimination because of intraspecific variation, it is useful in some cases, including for the dorsal, sternal and ventrianal shields (compare *Zygoseius papaver* and *Zygoseius lindquisti*, Figs 1–2, 28–29, 15–16, 32–33; Halliday 1997).

The epistome and the male chelicerae appear as the most studied (or most often illustrated) gnathosomal characters in *Zygoseius*. There is some interspecific variation in the epistome, including the number (usually 2, rarely 3 or 4) and length of projections, and the extent of barbs on the margins. These variations are overall only moderate, although overall represent useful diagnostic features. Male chelicerae may be useful, with some apparent variation in dentition and in the lengths of spermatodactyls (e.g. *Zygoseius furciger* has a longer spermatodactyl relative to cheliceral digits; Halliday 1997, Karg 1998, Karg and Schorlemmer 2009). The dentition of the female chelicerae has been illustrated for a few species only (*Zygoseius incisus*, *Zygoseius alveolaris*, *Zygoseius furciger* (in Halliday 1997), *Zygoseius papaver* sp. n.), and may differ in some species (e.g. *Zygoseius incisus* has stronger teeth). The deutosternum has a variable numbers of transversal rows of denticles; e.g. that of *Zygoseius papaver*, *Zygoseius lindquisti* and *Zygoseius furciger* have 7, 6–7 and 8 rows of denticles, respectively. The relative lengths of hypostomal setae (*h1*–*h3*, *pc*) also vary significantly, with some species having a particularly long *h1* seta (e.g. in *Zygoseius papaver* sp. n.), whereas in other species (e.g. *Zygoseius lindquisti* sp. n., *Zygoseius furciger*), *h3* tends to be the longest.

**Figure 27.**
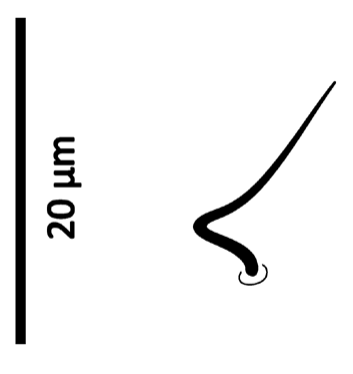
Seta *av* on palp trochanter of *Zygoseius papaver* sp. n., *Zygoseius lindquisti* sp. n. and *Zygoseius furciger*.

**Figure 28.**
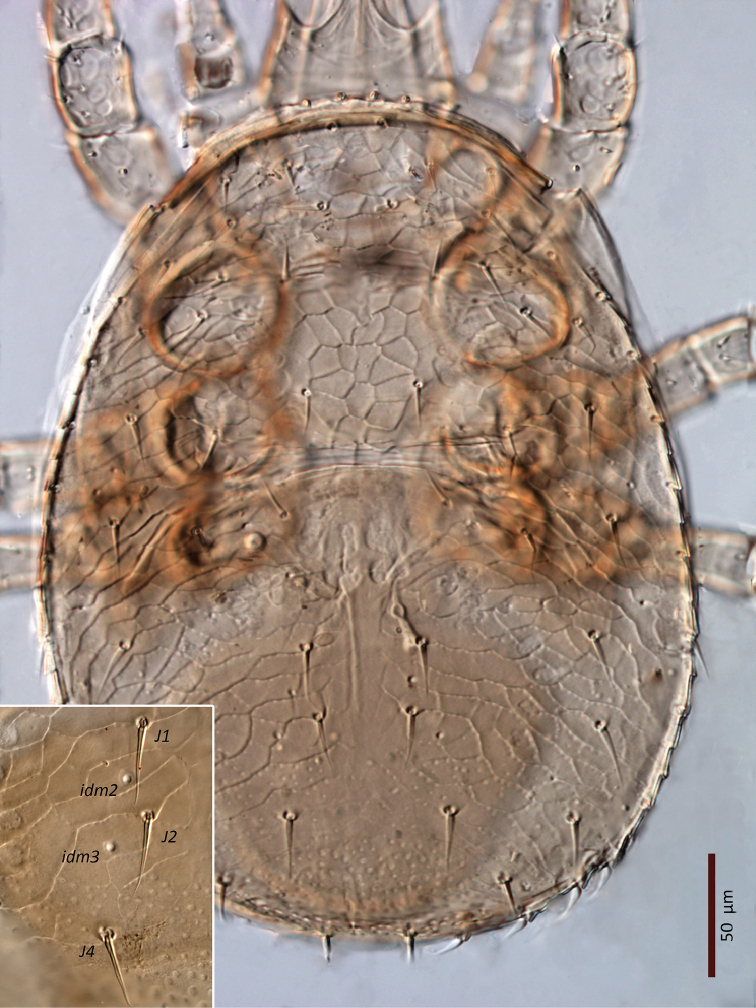
*Zygoseius papaver* sp. n., female, dorsal idiosoma.

**Figure 29.**
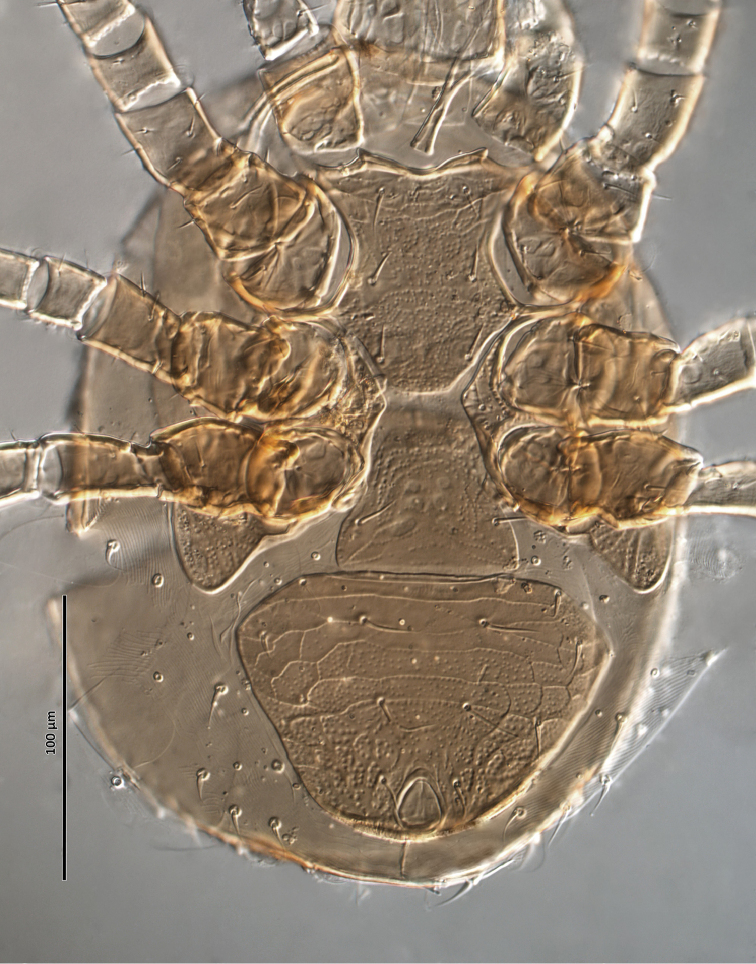
*Zygoseius papaver* sp. n., female, ventral idiosoma.

**Figure 30.**
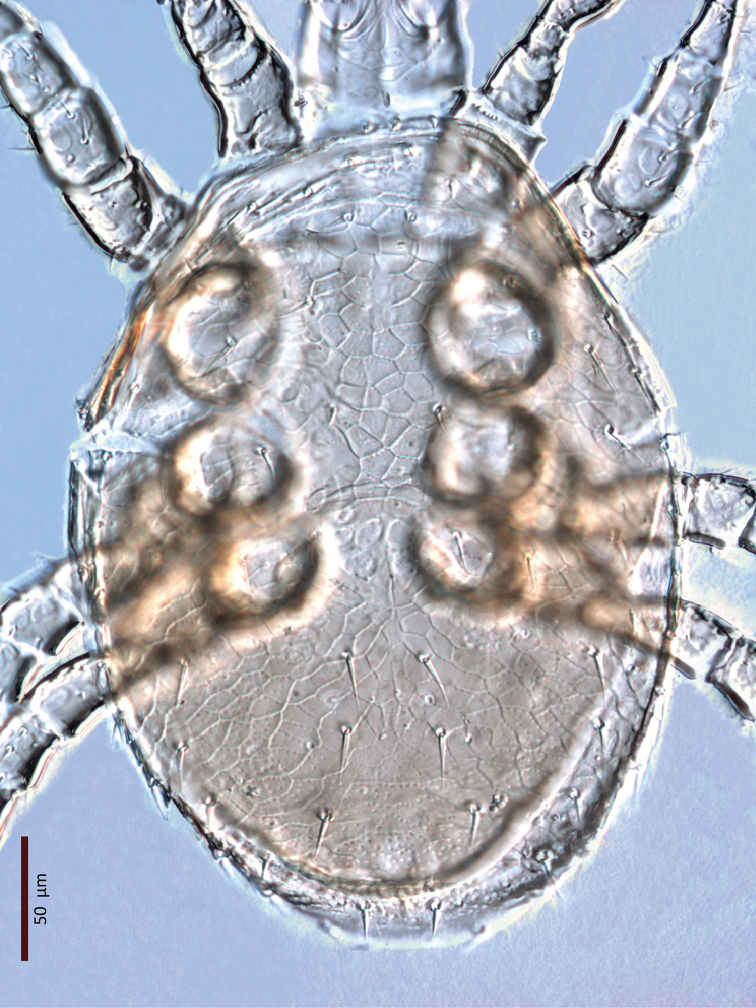
*Zygoseius papaver* sp. n., male, dorsal idiosoma.

**Figure 31.**
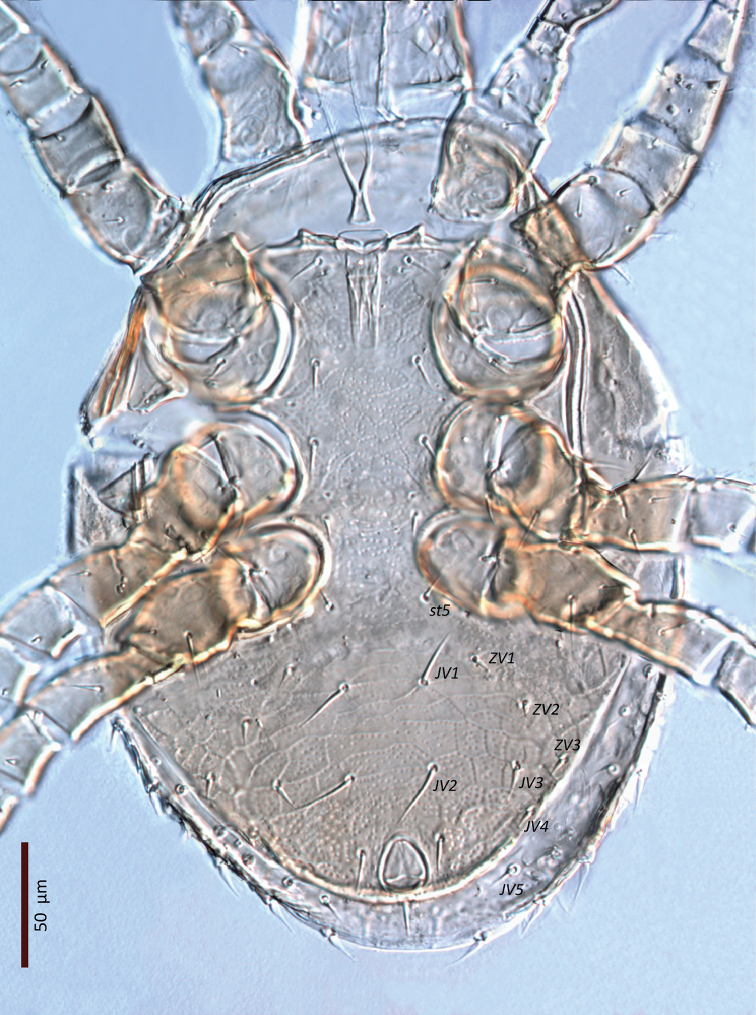
*Zygoseius papaver* sp. n., male, ventral idiosoma.

**Figure 32.**
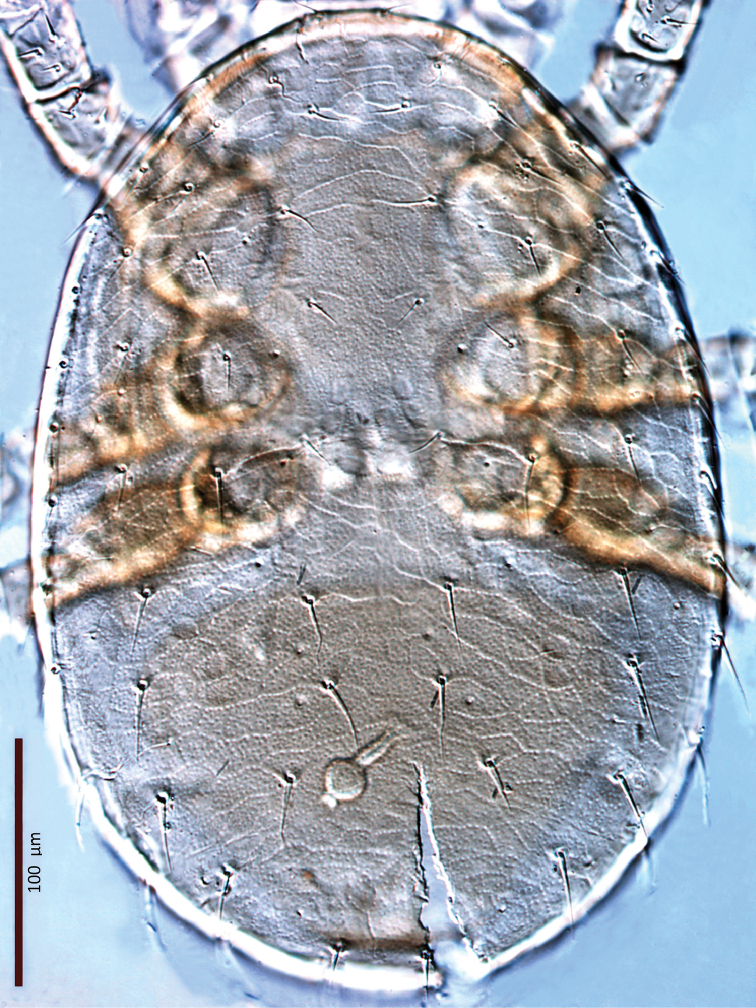
*Zygoseius lindquisti* sp. n., female, dorsal idiosoma.

**Figure 33.**
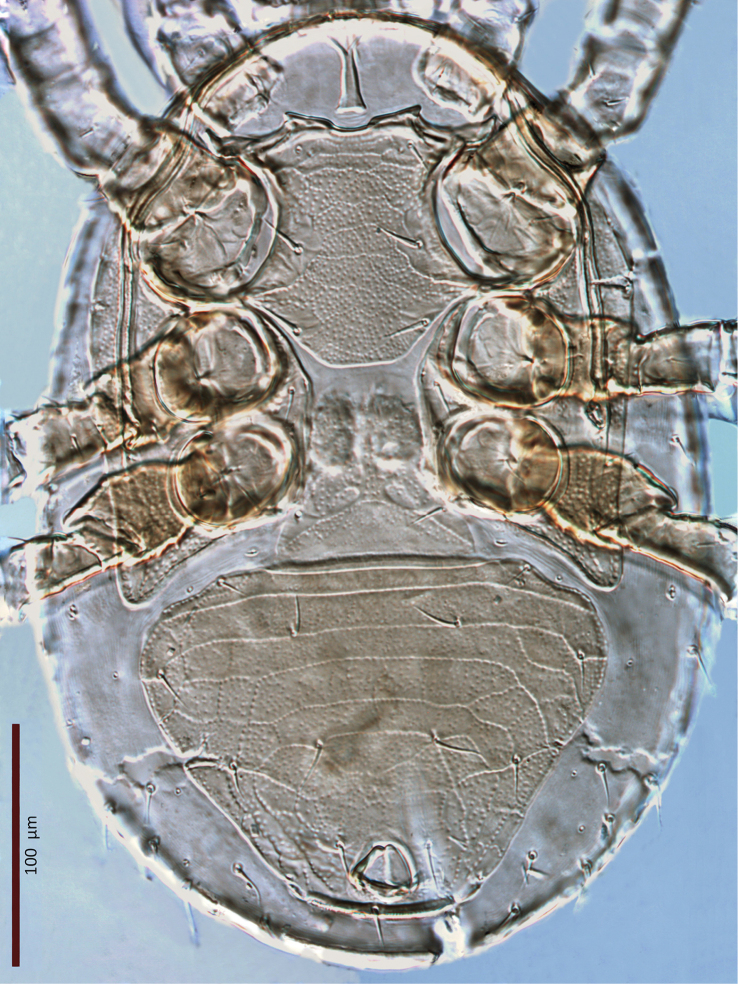
*Zygoseius lindquisti* sp. n., female, ventral idiosoma.

## Supplementary Material

XML Treatment for Zygoseius papaver

XML Treatment for Zygoseius lindquisti
